# Plasma metabolomics by nuclear magnetic resonance reveals biomarkers and metabolic pathways associated with the control of HIV-1 infection/progression

**DOI:** 10.3389/fmolb.2023.1204273

**Published:** 2023-06-29

**Authors:** León Gabriel Gómez-Archila, Martina Palomino-Schätzlein, Wildeman Zapata-Builes, Maria T. Rugeles, Elkin Galeano

**Affiliations:** ^1^ Grupo de Investigación en Sustancias Bioactivas, Facultad de Ciencias Farmacéuticas y Alimentarias, Universidad de Antioquia (UdeA), Medellín, Colombia; ^2^ Grupo de Investigación en Ciencias Farmacéuticas ICIF-CES, Facultad de Ciencias y Biotecnología, Universidad CES, Medellín, Colombia; ^3^ Servicio de RMN, Centro de Investigación Príncipe Felipe, Valencia, España; ^4^ Grupo Inmunovirología, Facultad de Medicina, Universidad de Antioquia (UdeA), Medellín, Colombia; ^5^ Grupo Infettare, Facultad de Medicina, Universidad Cooperativa de Colombia, Medellín, Colombia

**Keywords:** NMR, metabolomics, HIV-1, biomarkers, pathways

## Abstract

How the human body reacts to the exposure of HIV-1 is an important research goal. Frequently, HIV exposure leads to infection, but some individuals show natural resistance to this infection; they are known as HIV-1-exposed but seronegative (HESN). Others, although infected but without antiretroviral therapy, control HIV-1 replication and progression to AIDS; they are named controllers, maintaining low viral levels and an adequate count of CD4^+^ T lymphocytes. Biological mechanisms explaining these phenomena are not precise. In this context, metabolomics emerges as a method to find metabolites in response to pathophysiological stimuli, which can help to establish mechanisms of natural resistance to HIV-1 infection and its progression. We conducted a cross-sectional study including 30 HESN, 14 HIV-1 progressors, 14 controllers and 30 healthy controls. Plasma samples (directly and deproteinized) were analyzed through Nuclear Magnetic Resonance (NMR) metabolomics to find biomarkers and altered metabolic pathways. The metabolic profile analysis of progressors, controllers and HESN demonstrated significant differences with healthy controls when a discriminant analysis (PLS-DA) was applied. In the discriminant models, 13 metabolites associated with HESN, 14 with progressors and 12 with controllers were identified, which presented statistically significant mean differences with healthy controls. In progressors, the metabolites were related to high energy expenditure (creatinine), mood disorders (tyrosine) and immune activation (lipoproteins), phenomena typical of the natural course of the infection. In controllers, they were related to an inflammation-modulating profile (glutamate and pyruvate) and a better adaptive immune system response (acetate) associated with resistance to progression. In the HESN group, with anti-inflammatory (lactate and phosphocholine) and virucidal (lactate) effects which constitute a protective profile in the sexual transmission of HIV. Concerning the significant metabolites of each group, we identified 24 genes involved in HIV-1 replication or virus proteins that were all altered in progressors but only partially in controllers and HESN. In summary, our results indicate that exposure to HIV-1 in HESN, as well as infection in progressors and controllers, affects the metabolism of individuals and that this affectation can be determined using NMR metabolomics.

## Introduction

Human immunodeficiency virus type 1 (HIV-1), the causal agent of the acquired immunodeficiency syndrome (AIDS) in humans (Barré-Sinoussi et al., 1983; Gallo et al., 1983), continues to be a serious public health problem, after nearly 40 years of research (ONUSIDA and Comunicaciones y Promoción Mundial, 2021), generating considerable mortality among those infected, and an excessive cost for the healthcare system (Roth et al., 2018; Frank et al., 2019).

The progression of infection from the acute phase to advanced infection or AIDS is a very complex process, which takes approximately 10 years in absence of treatment (Vergis and Mellors, 2000; Kumar, 2013). Some individuals can naturally control HIV-1 replication, maintaining low viral load (VL) levels and an adequate count of CD4^+^ T lymphocytes, in the absence of antiretroviral therapy (ART) for at least 1 year (Cao et al., 1995; Baker et al., 2009). These individuals are known as controllers (elite or viremic) and exhibit specific resistance mechanisms to disease progression, including the presence of HLA alleles, HLA-B27 and HLA-B57 (Gonzalo-Gil et al., 2017).

Likewise, researchers have tried to characterize the natural resistance to HIV-1 infection among people exposed to the virus, who remain seronegative, known as HIV-exposed seronegative (HESN) individuals (Meyers and Fowke, 2010; Young et al., 2011). To date, only the homozygous *Δ32* mutation in the *CCR5* gene, the main entry coreceptor of the virus, has been consistently associated with host resistance to HIV-1 in less than 3% of resistant individuals (Huang et al., 1996; Ding et al., 2021). Other known genetic and immunologic factors involved in resistance to HIV-1 infection only partially explain this phenomenon (Lederman et al., 2010; Taborda-Vanegas et al., 2011), which means that further mechanisms remain unclear.

In this context, metabolomics understood as the objective identification and quantification of small molecules in biological fluids (Nicholson et al., 1999), might help to understand the biochemical state of an organism for discovering biomarkers. Through case-control studies of metabolites in plasma, urine, or cells, by quantitative measurement using nuclear magnetic resonance (NMR) spectroscopy, different pathophysiological states have been explained (Bertini et al., 2012; Li and Deng, 2016; Paris et al., 2018), suggesting that metabolomics could be a potential tool for prognosis, diagnosis, and monitoring the efficacy of treatment (Puchades-Carrasco and Pineda-Lucena, 2015), including HIV-1 infection.

Studies of the HIV effects on metabolism during *in vitro* replication and infection in animal and human models have provided new insights and targets for biomarker development and therapy. To date, little is known about the metabolic profiles that generate resistance to infection or a differential response to AIDS and its progression.

In the current study, we hypothesize that differences in the phenotype of infected individuals with high or low viral loads and seronegative individuals continuously exposed to HIV-1 will result in a dissimilar metabolomic plasma profile. Therefore, we collected and analyzed plasma samples of age and sex-matched groups of progressors, controllers, HESN and healthy controls by proton Nuclear Magnetic Resonance Spectroscopy (^1^H NMR). We aim to identify a specific metabolic fingerprint of each group and to obtain biomarkers related to HIV-1 progression and natural resistance, providing valuable information on the pathogenesis of HIV-1 infection.

## Materials and methods

### Chemicals and materials

All solvents and reagents were analytical grade, sodium phosphate dibasic dihydrate, sodium azide, deuterium oxide, 3-(Trimethylsilyl) propionic-2,2,3,3-d_4_ acid sodium salt (TSP-d_4_) and 3-(Trimethylsilyl)-1-propanesulfonic acid-d_6_ sodium salt (DSS-d_6_) were supplied by Merck (Germany). The ultrapure water was obtained in a Milli-Q purification system of Merck Millipore. The Vivaspin^®^ 500 3000 K MWCO Centrifugal Concentrators were provided by Sartorius.

### Human subjects

A retrospective cross-sectional study was developed using a defined database of volunteers to build case-control relationships. Plasma samples from 88 volunteers were evaluated distributed as follows:

• HESN: thirty individuals, from serodiscordant couples (couples in which one partner is HIV-positive and the other HIV-negative). HESN reported multiple unprotected sexual episodes for >2 years at the time of enrollment, with at least five episodes of at-risk intercourse within 6 months before study entry with an HIV positive partner with a detectable viral load (Zapata et al., 2008). The median VL of the partner was 2,569 RNA copies/mL (interquartile range = 400–25,250 copies/mL) (Aguilar-Jiménez et al., 2013). From these individuals, 10 (35%) were ART-naïve [VL median (interquartile range)] [10,257 (718–23,188)]. Eight (25%) were ART-responders VL < 400. Finally, 12 (40%) were ART-non-responders [ (Fisher et al., 1987), 806 (18,200–118,770)]. No Δ32-homozygous subjects were included.
• Controllers: fourteen, with 1 year of diagnosis of HIV-1 infection, and viral load less than 2000 copies/mL in the absence of Antiretroviral therapy (ART) and normal CD4^+^ T lymphocyte count (Pereyra et al., 2008). The median diagnosis time was 46 months (range 12–168). The median VL was 211 copies/mL (range 20–1885), and the median CD4^+^ T cells count was 745 cells/uL (range 514–1,367). Only 2 (14%) controllers showed the HLA-B*27 allele, and 3 (21%) controllers showed the HLA-B*57 allele.
• Chronic progressors: fourteen, with a CD4 + T lymphocyte count> 350 cells/μL and a viral load between 10,000 and 100,000 copies/mL without receiving ART (Taborda et al., 2015). The median diagnosis time was 51 months (range 12–120). From these, ten individuals had between 1 and 5 years of infection, three reported 6–9 years, and one had 10 years of infection. The median VL was 31,552 copies/mL (range 11,206–160405) and the median CD4^+^ T cells count was 443 cells/uL (range 267–819).
• PLHIV: people living with HIV. In this case it refers to a mix of controllers and progressors.
• Healthy controls: thirty, with negative serological tests for HIV-1 without risk behaviors.

This study was approved by the Bioethical Committee Universidad de Antioquia; and all the individuals signed informed consent prepared according to Colombian Legislation Resolution 008,430/1993.

### Processing of blood samples

The Blood sample was collected from all participants by using potassium-EDTA collection tubes. Then, it was centrifuged at 1,000 x g for 5 min at 4°C, and 2 mL of plasma was stored at −80°C until processing. Two methodologies were established to process the biofluid: a direct analysis and a deproteinization analysis.

For the direct analysis 300 µL of Buffer pH 7.4 (Na_2_HPO_4_ 75 mM DSS 2.3 mM and NaN_3_ 0.04%) was added to a microcentrifuge tube (1.5 or 2 mL) and reserved. Then, the plasma sample was thawed, homogenized, and 300 µL transferred to the previously mentioned vial with buffer. The resulting solution was mixed and 550 µL transferred to a 5 mm NMR tube for analysis. Tubes were degassed for 3 min before capping.

In the case of deproteinization analysis, 300 µL of Buffer pH 7.4 (Na_2_HPO_4_ 75 mM TSP 2.3 mM and NaN_3_ 0.04%) was added to a microcentrifuge tube (1.5 or 2 mL) and reserved. Then, the vial containing the sample was thawed, the plasma was homogenized, and 500 µL of plasma were taken to a Vivaspin 500 centrifugal filter (Previously pre-washed 5 times with Buffer pH 7.4). The sample was centrifuged at 12,000 gravities at 4°C for 60 min 300 µL of the filtrate was brought to the microcentrifuge tube containing the 300 µL of Buffer pH 7.4 they were mixed with a micropipette. Finally, 550 μL of the solution were taken and transferred to a 5 mm NMR tube for analysis. The tubes were degassed for 3 min before being capped.

### ^1^H-NMR experiments

The ^1^H-NMR spectra of extracts were recorded at 300 K by a Bruker AVANCE III 600.13 MHz spectrometer equipped with 5 mm triple-resonance z-gradient cryoprobe (Prodigy TCI 1H-13C/15N-2H). TopSpin version 3.6.2 (Bruker GmbH Karlsruhe Germany) was used for spectrometer control purposes. Carr-Purcell-Meiboom-Gill (CPMG) pulse sequence with water presaturation and spoil gradients (*cpmgpr1d* pulse sequence) for direct analysis (64 k data points, spectral width 12,019 Hz, dummy scans 8, 64 scans, loop for T2 filter 80, gain 80,6, delay time 4 s, fixed echo time 0.0007 s and the 90° pulse length was adjusted to about 10.40 µs). ^1^H 1D Nuclear Overhauser Effect Spectroscopy (NOESY) NMR spectra with water presaturation and spoil gradients (*noesygppr1d* pulse sequence) was used for analysis of deproteinization samples Spectra were acquired with (Thomsen et al., 2011) scans, 64 k data points, spectral width of 7,211 Hz, and relaxation delay of 20 s (dummy scans 4, gain 203, and the 90° pulse length was adjusted to about 10.42 µs)

Total Correlation Spectroscopy (TOCSY) and multiplicity Heteronuclear Single Quantum Correlation (HSQC) were performed on representative samples with 256–512 t1 increments 32–96 transients and a relaxation delay of 1.5 s. The TOCSY spectra were recorded by a standard MLEV-17 pulse sequence with mixing times (spin-lock) of 65 ms.

## Data analysis and statistics

**NMR spectra processing:** ^1^H-NMR spectra were transformed with a 0.5 line-broadening and manually baseline- and phase-corrected with Topspin 3.6.4. NMR signals of DSS-d6 (for direct analysis) or TSP-d_4_ (for deproteinization analysis) were referenced to 0.0 ppm. For metabolite identification purposes the ^1^H and chemical shift values and multiplicity of signals were compared with the reference data from the Chenomx software (Chenomx NMR Suite 8.4 Chenomx Inc. Edmonton Canada) in combination with spectral databases Human Metabolome Database, and the Biological Magnetic Resonance Bank and several literature reports (Ulrich et al., 2007; Wishart et al., 2018). Optimal integration regions were defined for each metabolite to select signals without overlapping. Integration was performed with MestreNova 14 (Mestrelab Research SL Santiago de Compostela Spain) by manually integrating of the previously identified signals. With these regions an integration matrix (Integral Regions) was built which was later applied to the 88 acquired spectra and a matrix of integrals was built for all the spectra (Integral series). This matrix of integrals was normalized by the sum of the total signals of the spectrum using Excel (Microsoft United States of America).

**Multivariate and univariate analysis of Metabolomic Profiles:** The previously normalized matrix of integrals was processed using MetaboAnalyst 5.0. First a principal component analysis (PCA) was performed which allowed finding groups of samples with a similar metabolic pattern and/or segmenting those with a different metabolome.

Then five case-control relationships were established: healthy controls *versus* progressors; healthy controls *versus* controller; healthy controls *versus* PLHIV (progressors and controllers); healthy controls *versus* HESN, and controllers *versus* progressors. These relationships were evaluated by Partial Least Squares Discriminant Analysis (PLS-DA) which links two data matrices and improves the separation between different groups of samples. The quality of the PLS-DA was evaluated, and a permutation test was carried out to calculate the goodness of fit (R2) and the predictive capacity (Q2) of the randomly generated models. An analysis of the results of the PLS-DA statistic (VIP scores) was performed and the metabolites that contributed significantly to the separation of the groups were identified. Variables with a VIP score greater than 1.0 were considered significant for the model.

Finally, the selected variables were subjected to a Univariate analysis using a difference of means test (Wilcoxon Test). For tests with a *p*-value less than 0.05 (*p <* 0.05) a statistically significant difference between the means (mean or median as appropriate) was assumed for the variable evaluated. In the case of obtaining more than one significant signal for a given metabolite, we selected the signal with less overlapping for graphical representation.

### Gene analysis

Analysis of associated genes was carried out with the metabolites that were statistically significant after univariate analysis. For this, the web interface of MetaboAnalyst 5.0 [14] was used. Metabolites were introduced in the *Network Explorer* section of the platform and the *Metabolite-Gene-Disease Interaction Network analysis* was carried out, which provides a global view of potential functional relationships between metabolites, connected genes, and target diseases. The network integrates gene-metabolite, metabolite-disease, and gene-disease interaction networks.

The genes identified through the previous analysis were filtered through a comparison process with the National Center for Biotechnology Information (NCBI) gene database of the U.S. National Library of Medicine, excluding genes unrelated to HIV-1. Additionally, to perform a more specific analysis, genes that were not related to two or more groups and/or metabolites were excluded.

Then, we proceeded to perform an individual analysis of the selected genes through a review in the NCBI gene database. Looking specifically at the section on HIV-1 interactions, we filter further into the subcategories *Replication interactions* (human proteins shown to be required for HIV-1 infectivity and replication) and *Protein interactions* (proteins that have been shown to interact with proteins from HIV-1).

## Results

### Human subjects

**FIGURE 1:**
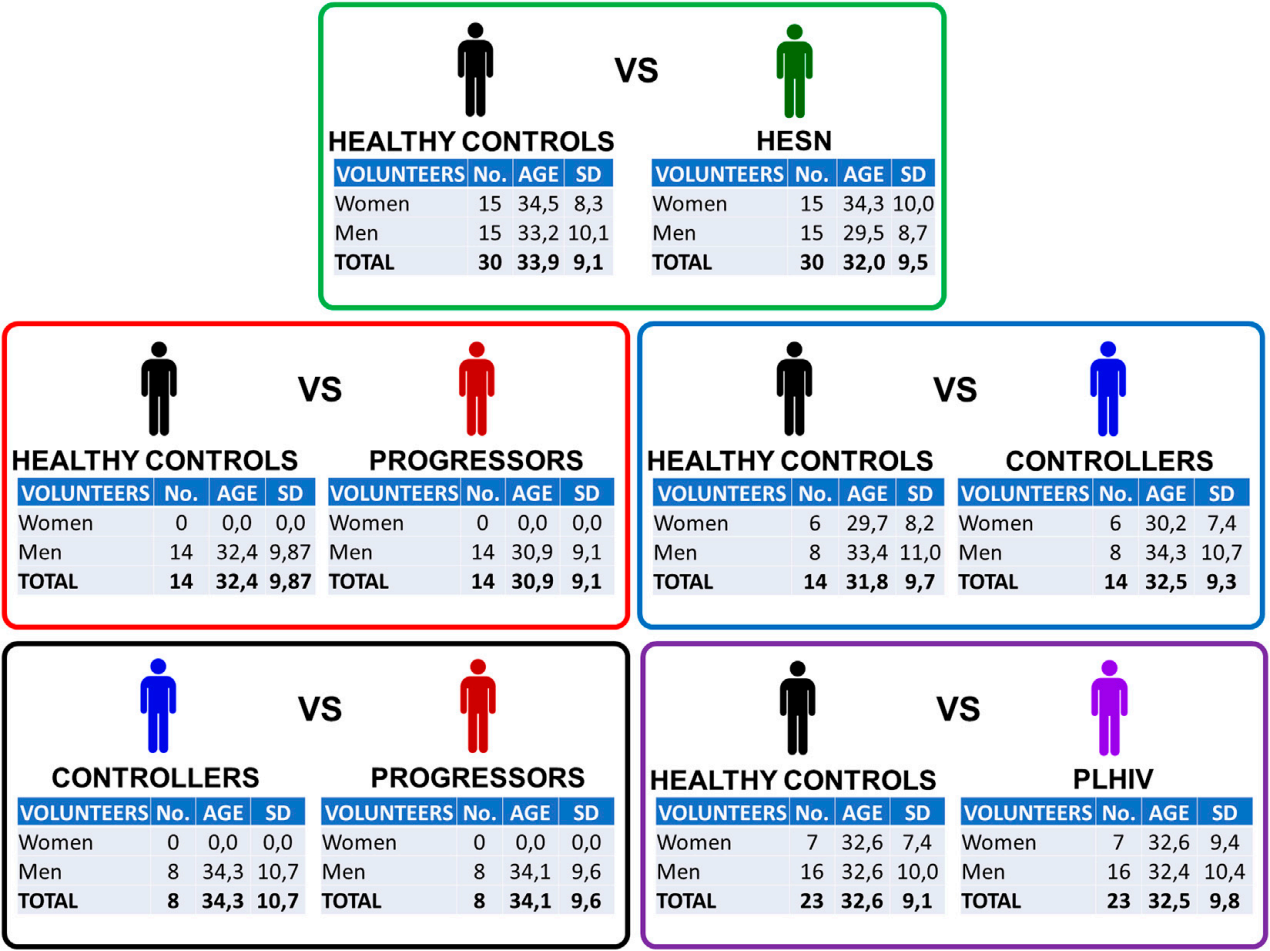
Case-control relationships. Detail of each of the five relationships built to analyze plasma samples in a direct and filtered way. To know the details of each of the volunteers who participated in the study, see Supplementary Table S1. Exclusion criteria: Individuals with hemoglobin ≤8.0 g/dl; neutrophil count ≤1,000/mm3; receiving some immunosuppressive treatment; pregnant or lactating women; cancer or with an active infection or disease requiring hospitalization. Age reported as the sample mean. SD: standard deviation.

Figure 1 shows the main characteristics of the five case-control relationships analyzed in the study. Out of the 88 available volunteers, a selection was made to generate groups balanced by gender and age. A summarized table of all individuals can be found in Supplementary Table S1

To compare HESN with healthy individuals, we achieved a completely gender-balanced comparison, with 15 women and 15 men in each group. Also, in the case of controllers, the number of women and men was similar in both groups. In the case of joining controllers and progressors (PLHIV), we built up groups with a higher number of men than women, but that was still gender-matched between patients and controls.

### Analysis of metabolic plasma profiles

^1^H NMR metabolomics analysis was performed on intact and filtered plasma samples, to identify the highest possible number of compounds. During the spectral analysis process, it was possible to identify fifty-five metabolites in the direct plasma samples: two alcohols, twenty-one amino acids, fourteen lipid-related signals, sixteen organic acids, one purine derivative, and one sugar. In the filtered plasma samples, forty-five metabolites were identified: two alcohols, twenty amino acids, two lipid-related signals, eighteen organic acids, two derived from purine and one from sugar. It should be noted that three metabolites that were not observed in direct plasma could be detected in filtered plasma samples: two organic acids (2-hydroxybutyrate and 3-Hydroxyisovalerate) and one purine derivative (Inosine). In Supplementary Figures S1, S2 a model NMR spectrum can be seen with the relative assignment for direct and filtered Plasma respectively. Furthermore, the quantification of small metabolites was more accurate in the filtered samples due to the absence of overlapping with broad lipoprotein samples. It should be noted that the lipoproteins evaluated in the unfiltered samples complement the metabolomic analysis of the plasma samples from the volunteers. To know the details of the metabolites identified in the plasma samples, see Supplementary Table S2.

After assignment, normalized integration tables were obtained of all spectra (Supplementary Table S3) and analyzed by multivariate analysis.

### Multivariate analysis of plasma metabolomic profiles

**FIGURE 2:**
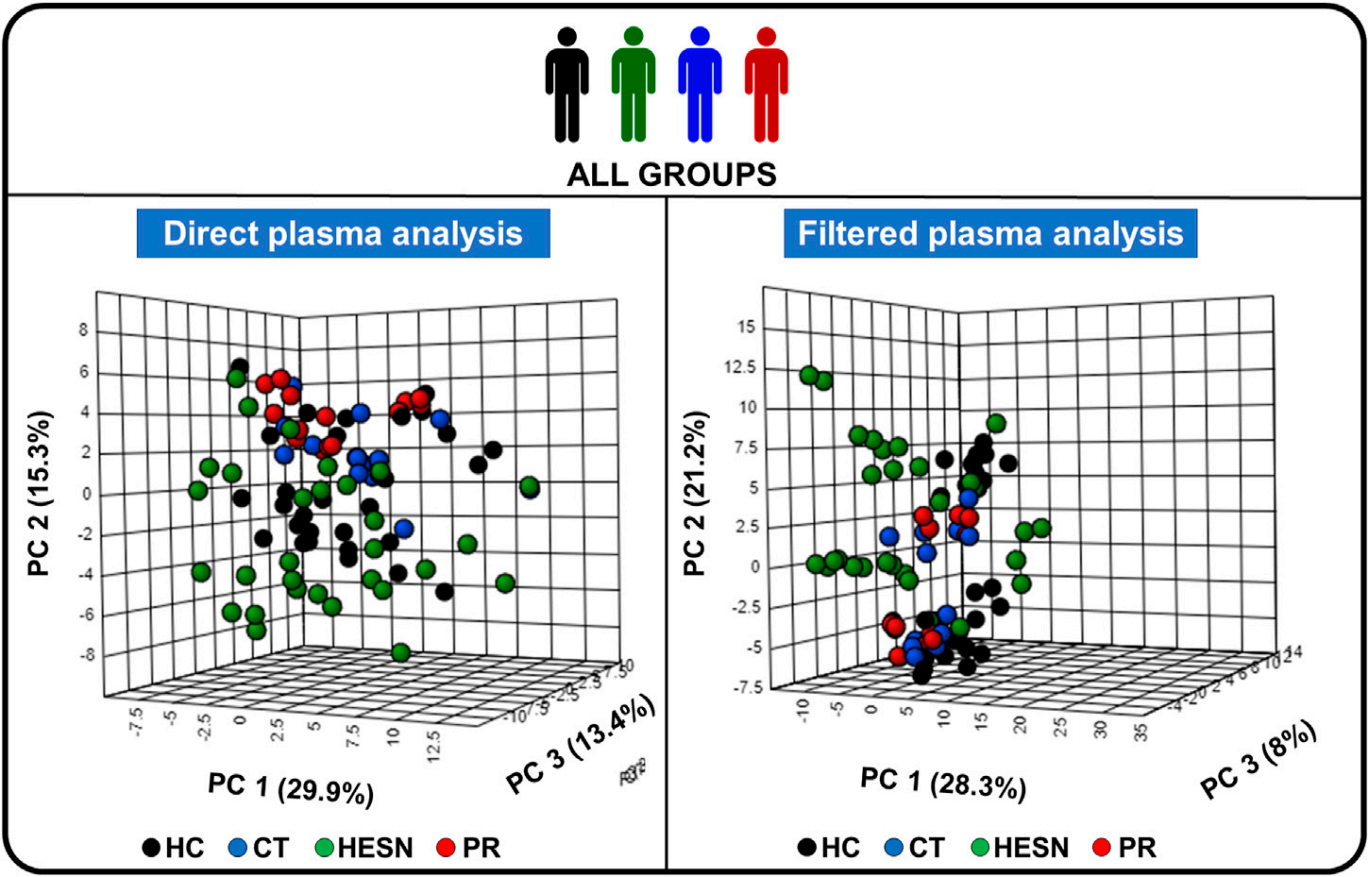
Principal component analysis (PCA) of the study groups. 3D score plot charts of the PCA analysis of all study volunteers, on the left side the analysis with direct plasma and on the right side with filtered plasma. PC: Principal component, HC: Healthy control, CT: controller, HESN: HIV-exposed seronegative, PR: Progressor. Each of the three axes of the graph represents a principal component. The values that each of the axes takes is related to the fact that there is a score value for each observation (row) in the data set; so, there is score values for the first component, another for the second component, and one for the third. The score value for an observation, say the first component, is the distance from the origin, along the direction (load vector) of the first component, to the point where that observation projects onto the direction vector.

Initially, a principal component analysis (PCA) was performed with all 88 samples to get a general overview. The result is shown in Figure 2, which corresponds to the PCA score plots of direct plasma samples and filtered plasma samples. In this unsupervised analysis, we detected a clustering between samples belonging to the same group (healthy control, controller, HESN or progressor). This grouping was more evident for direct plasma samples, which could indicate that lipoproteins play a key role in the differentiation of the groups. HESN samples seem to have the highest dispersion. PCAs were also performed for the five established case-control relationships, which can be seen in Supplementary Figure S3.

**FIGURE 3:**
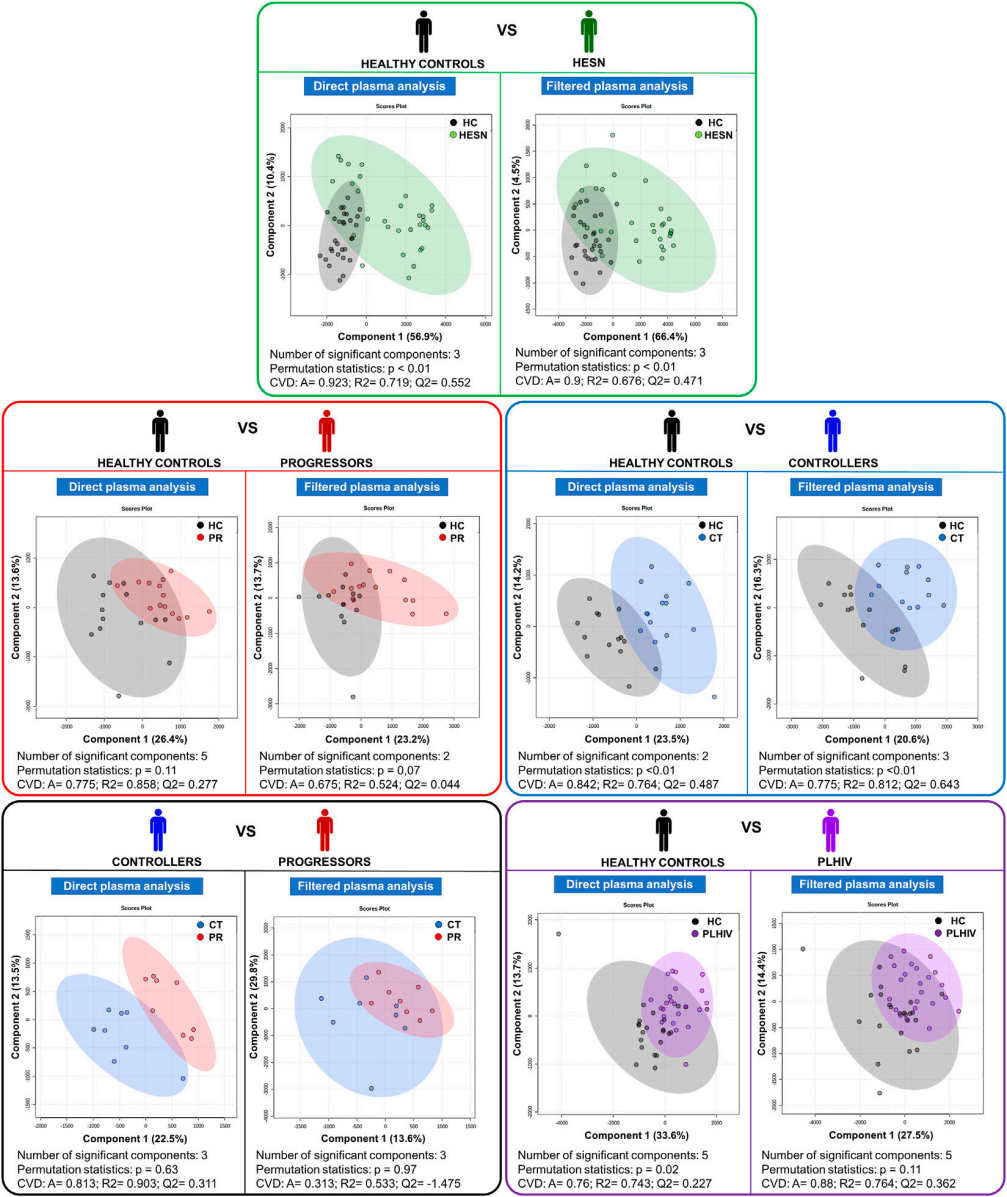
Partial Least Squares Discriminant Analysis (PLS-DA). Score plot charts of the PLS-DA of the five case-control relationships established; on the left side, the analysis with direct plasma and on the right side with filtered plasma (in each colored box). HC: Healthy control, CT: controller, HESN: HIV-exposed seronegative, PR: Progressor. CVD: Cross-validation details, A: Accuracy, R2: goodness of fit, Q2: predictive capacity. Healthy controls in black, HESN in green, Progressors in red, Controllers in blue and PLHIV in purple.

In addition, to observe specific metabolic differences between our five case-control relationships, a pair-wise Partial Least Squares Discriminant Analysis (PLS-DA) was performed (see Figure 3).

Statistically significant PLS-DA models were obtained comparing HESN and controllers with healthy controls. Models between healthy controls and progressors and healthy controls *versus* PLHIV were statistically significant. The analysis of the PLS-DA statistics through the VIP score allowed reducing the number of relevant variables (metabolites) to be analyzed as follows:

• Healthy controls *versus* HESN: 27 in direct plasma (DP) and 26 in filtered plasma (FP).
• Healthy controls *versus* progressors: 47 in DP and 33 in FP.
• Healthy controls *versus* controllers: 35 in DP and 38 in FP.
• Healthy controls *versus* PLHIV (progressors and controllers): 39 in DP and 36 in FP.
• Controllers *versus* progressors: 45 in DP and 39 in FP.

For the details of the PLS-DA statistics see Supplementary Table S4.

### Univariate analysis of plasma metabolomic profiles

The metabolites relevant for PLS discriminant modeling were further submitted to univariate statistical analysis to identify significant changes in each case-control comparison. The detail of the mean difference analysis (Wilcoxon Test) can be seen in Supplementary Table S5.

For the Healthy controls *versus* HESN comparison, 21 signals (variables) were identified with a significant variation, 13 associated with the FP. These signals are associated with 13 metabolites: six lipid-related signals (Low density lipoprotein aliphatic chain, Low density lipoprotein (CH3), very low-density lipoprotein (CH3), Lipids CH2C = C, lipids (-n (CH3)3 and VDL (aliphatic chain)), three amino acids (Serine, alanine and phosphocholine), two organic acids (3-Hydroxybutyrate and Lactate), one alcohol (Myo inositol) and one sugar (Glucose). Only the lactate was increased in HESN, the rest of the metabolites were decreased compared to healthy controls.

In healthy controls *versus* progressors comparison, 28 signals were identified, 19 associated with DP. These signals correspond to 14 metabolites: eight amino acids (Creatine, creatinine, Glutamine, Methionine, Serine, Alanine, Tyrosine and Valine), four lipid-related signals (Lipid: CH2CH2O, VLDL, Lipids CH2C = C, VDL-2 (aliphatic chain) and very low-density lipoprotein (CH3)), one alcohol (Myo inositol) and one sugar (Glucose). The metabolites of the lipid-related signals, Serine, Alanine and Tyrosine, are decreased.

While for the comparison healthy controls *versus* controllers 24 signals were identified (equal amount of each matrix), which were associated with 12 metabolites: five amino acids (Glutamine, Glutamate, Methionine, Serine and Valine), three lipid-related signals (Low density lipoprotein (CH3), very low-density lipoprotein (CH3) and lipids (-n (CH3)3), three organic acids (3-Hydroxybutyrate, Acetate and Pyruvate) and one sugar (Glucose).

**FIGURE 4:**
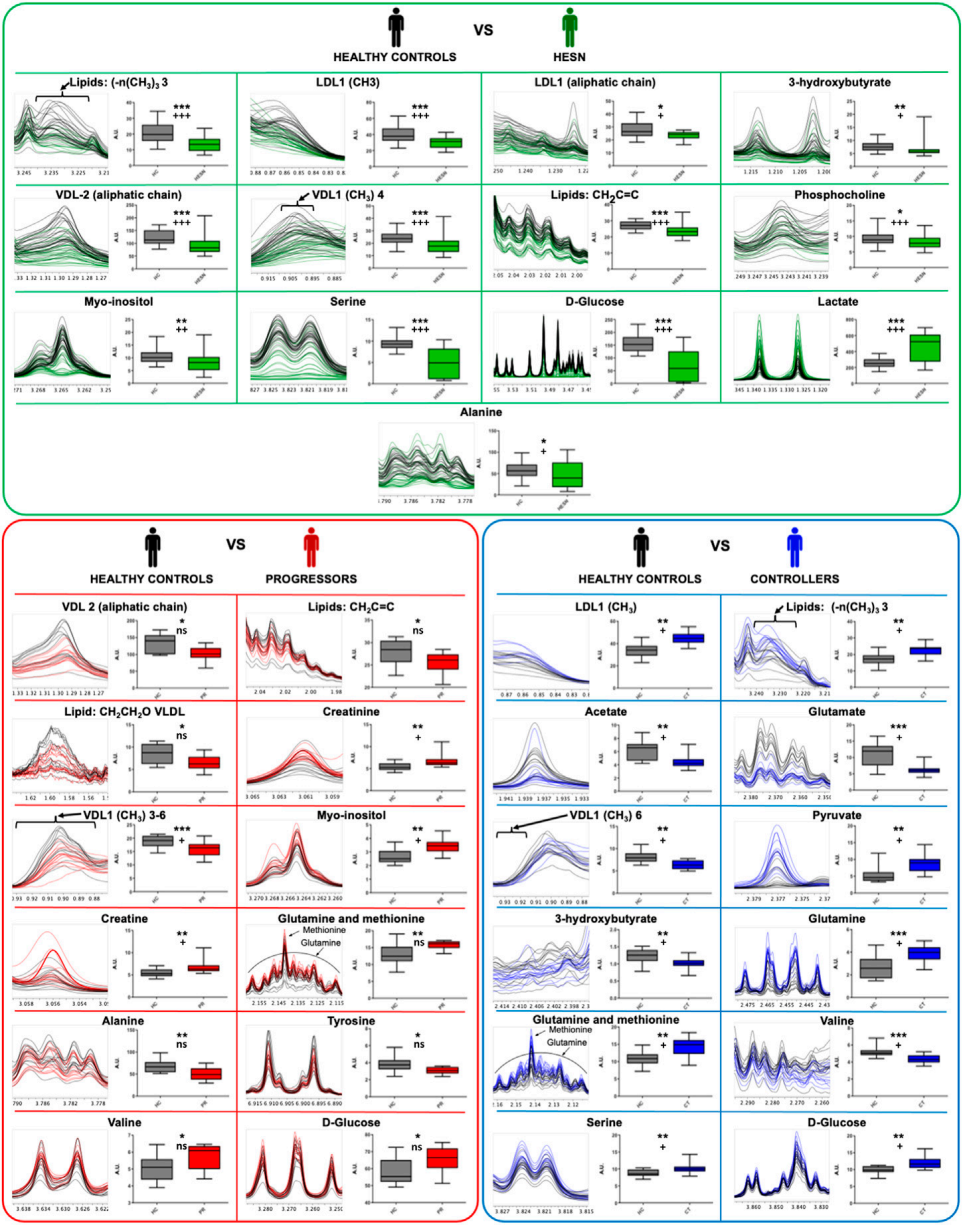
Metabolites that explain the difference between the groups. Results of the Wilcoxon test for the significant variables (metabolites) for each proposed PLS-DA model. In the panel **(A)** (main green box), the comparison of healthy controls and HESN, in the panel **(B)** (main red box) the comparison between healthy controls and progressives, and in the panel **(C)** (main blue box) the comparison between healthy controls and controllers. Within each small individual box: on the left the region of the spectrum (metabolite signal) 1H-NMR superimposed of all the samples analyzed in each comparison, on the right side the box-and-whisker plot for the normalized concentration and the statistical significance of each test. *: *p*-value < 0.05, **: *p*-value < 0.01 and ***: *p*-value < 0.001. +: False Discovery Rate (FDR) < 0.05, + +: FDR <0.01, + + +: FDR <0.001 and ns: FDR ˃0.05. Healthy controls in black, HESN in green, progressors in red, and the controllers in blue.

Figure 4 shows a quantitative comparison of the most representative metabolites that change between healthy controls and HESN, progressor and controller groups.

The comparisons of healthy controls *versus* PLHIV and controllers *versus* progressors can be seen in Supplementary Figure S4.

## Discussion

### All three study groups show a specific metabolic profile

**FIGURE 5:**
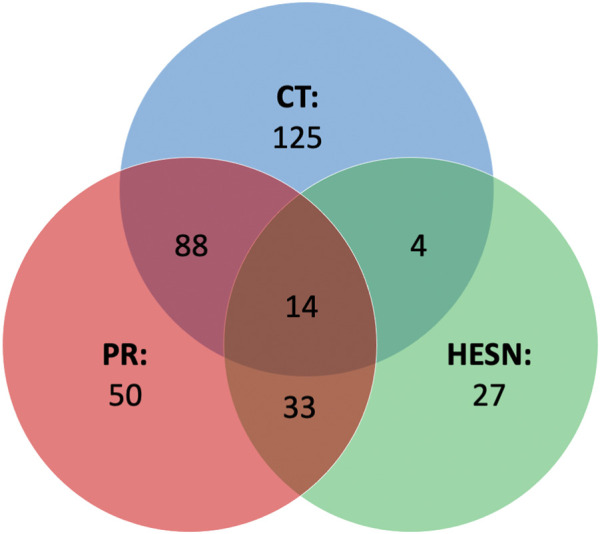
Venn diagram of metabolites that explain differences among groups. Set Analysis showing the metabolites related to differences similarities among the metabolomic profiles of controllers, progressors and HESN. In the main part of each set the metabolites related only to one study group and in the intercepts the metabolites related to two or more groups (central intercept). CT: controllers (In blue color set), HESN: HIV-exposed seronegative (In green color set), PR: Progressors (In red color set). HC: Healthy controls, 3-HB: 3-Hydroxybutyrate, ACE: Acetate, ALA: Alanine, CRE: Creatine, Cre: Creatinine, Glc: d-Glucose, GLN: Glutamine, GLU: Glutamate, LAC: Lactate, LDL1 (CH3): Low density lipoprotein (CH3), LDL (AC): Low density lipoprotein aliphatic chain, MET: Methionine, MYO: Myo-inositol, PHO: Phosphocholine, PYR: Pyruvate, SER: Serine, TYR: Tyrosine, UNK: Unknown, VAL: Valine, VDL (AC): VDL-2 (aliphatic chain), VDL1 (CH3): Very low-density lipoprotein (CH3), (-n (CH3)3: Lipids (-n (CH3)3, CH2CH2O VLDL: Lipid: CH2CH2O VLDL, CH2-C=C: Lipids CH2C = C.

PCA and the PLS-DA analysis demonstrated significant differences between the controllers, progressors, and HESN study groups *versus* healthy controls. In contrast, only weak models were obtained comparing controllers and progressors, which may be due to the low sample number in this case (n = 8). Differences were related to specific metabolites present in different concentrations between the groups as demonstrated by univariate analysis (Wilcoxon Test). For each case-control comparison, a list of metabolites with altered levels were established. Figure 5 provides an overview of all the important metabolites in the different comparisons.

**FIGURE 6:**
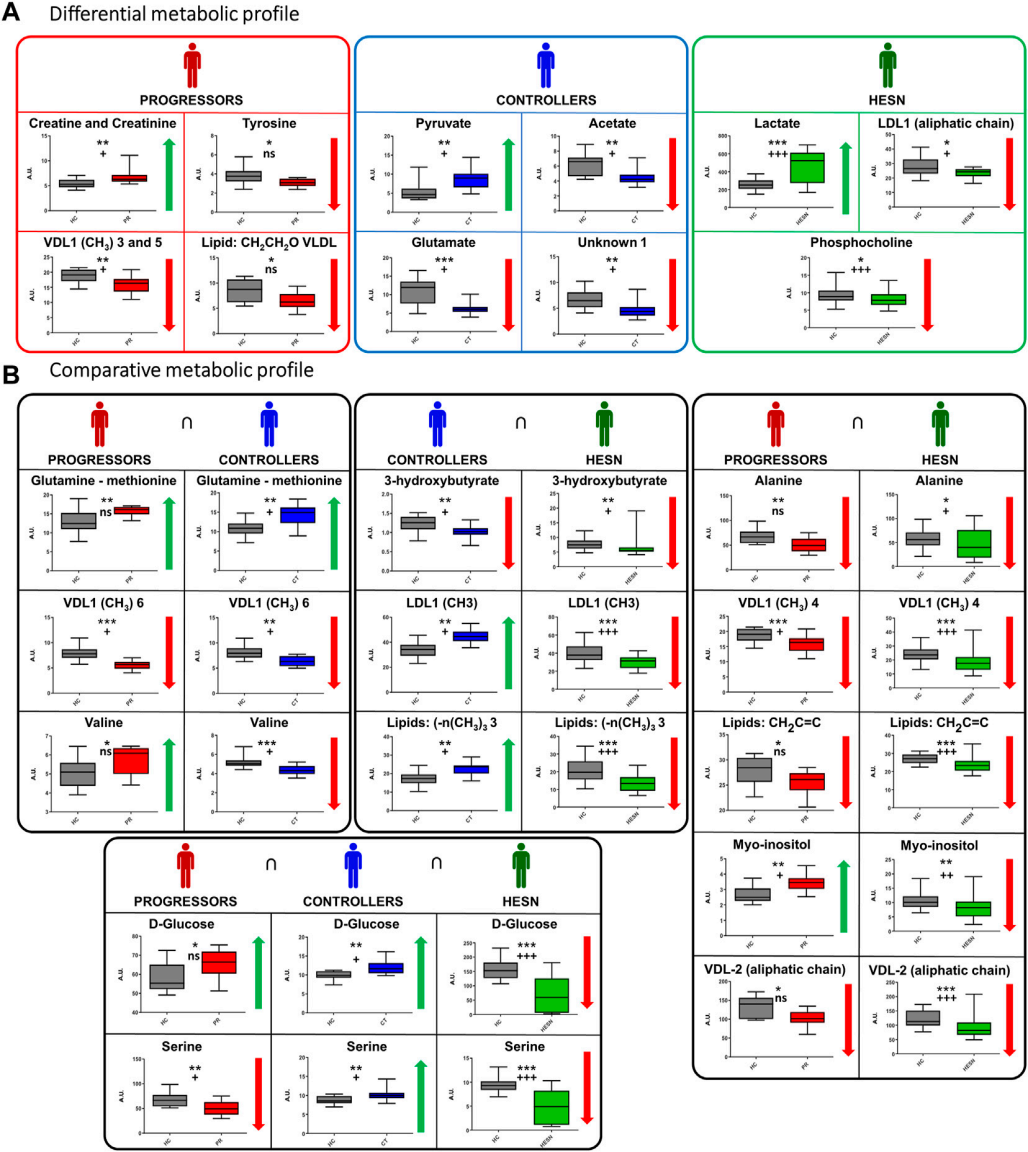
Specific and related metabolites in the study groups. **(A)** Differential metabolic profile: Metabolites that only exhibit a statistically significant mean difference compared to healthy controls in a study group, **(B)** Comparative metabolic profile: Metabolites exhibiting a statistically significant mean difference compared to healthy controls in two or more study groups. Variation: ↓ (Smaller area or relative concentration in the reference method) ↑ (Bigger area or relative concentration in the reference method). The box-and-whisker plot for the normalized concentration and the statistical significance of each test. *: *p*-value < 0.05, **: *p*-value < 0.01 and ***: *p*-value < 0.001. +: False Discovery Rate (FDR) < 0.05, + +: FDR <0.01, + + +: FDR <0.001 and ns: FDR ˃0.05. Healthy controls in gray, HESN in green, progressors in red, and the controllers in blue.

We observed that the disease altered the metabolomics blood profile of progressors, showing the highest number of relevant variables compared to healthy controls in the PLS-DA (47 in DP and 33 in FP), and the highest number of differentiated metabolites (Ding et al., 2021) in the univariate analysis. The impact on controllers was lower (35 variables in DP and 38 in FP, 12 relevant metabolites), while HESNs are the group with the lowest number of relevant variables in the PLS-DA (27 in DP and 26 in FP) and 13 differentiated metabolites. The progression of HIV induces the massive elimination of CD4^+^ T lymphocytes and alterations in various components of the immune system (Brenchley et al., 2006), which would explain the difference in the metabolic profiles of the progressors compared to controllers and HESN. Since the controllers resist the progression to AIDS, maintaining low levels of viral load and an adequate count of CD4^+^ T lymphocytes (Cao et al., 1995; Baker et al., 2009), it is directly reflected in a lesser impairment of their metabolism. Likewise, identifying of a differential metabolomic profile in HESNs allows us to affirm that the natural resistance of the host to HIV-1 is associated with a differential phenotype. Detailed analysis of the metabolomics changes (positive or negative variation) between the groups, as shown in Figure 6, allowed establishing differential (Figure 6A) and comparative profiles (Figure 6B) for each of the case-control comparisons.

***Alterations associated to progressors:*** Progressors stand out for the variation of specific lipoproteins and creatine/creatinine and tyrosine, while changes in glutamate, pyruvate and acetate seem to be specific for controllers. On the other hand, alterations in lactate, phosphocholine and VDL are characteristic of HESN.

Concerning the comparative analysis (Figure 6B), it is worth mentioning that glucose and serine change in all three case-control comparisons, although these changes do not always have the same sign. It is also noteworthy that progressors and HESN have five metabolic changes in common, four of which have the same sign.

Differentially, it was possible to identify an increase in the expression of creatine-creatinine in progressors; creatine is found in muscles (Kreider and Stout, 2021). Altered creatine-creatinine values have been previously found in HIV positive patients (Sitole et al., 2019) and are related to the prolonged period of high energy expenditure (Kosmiski, 2011) and cachexia (Von Roenn et al., 1992).

Likewise, tyrosine was decreased in the HIV progressors group. Tyrosine is a precursor of catecholamines (adrenaline, dopamine and noradrenaline) whose altered metabolism is related to mood disorders (Hasler et al., 2008). An increased phenylalanine/tyrosine ratio is common in patients with HIV-1 infection and is related to immune activation (Zangerle et al., 2010). Previous NMR studies identified tyrosine downregulation in untreated HIV-infected patients (Sitole et al., 2019). This change was not observed in HIV controllers, which allows us to state that low tyrosine levels are a biomarker of HIV infection progression.

One factor associated with HIV progression is the response of immune cells (Shi et al., 2022). Immune cells undergo energetic and structural remodeling following immune activation. It generates metabolic changes associated with increased energy and biosynthetic demands as viral load increases and the immune system responds (González Plaza et al., 2016). This metabolic changes, including lipid homeostasis, since mitochondria plays a key role in the biosynthesis of phospholipids for membranes, as well as in the catabolism of fatty acids (Tilokani et al., 2018). Unsurprisingly, phospholipid alterations are a common finding in the metabolic profiles of HIV-infected individuals.

A comparison of the commonly altered metabolites in controllers and progressors showed that valine is differentially regulated between both groups; it increased in progressors and decreased in controllers compared to healthy controls. A recent study show a significant increase in Valine levels in TEC before the loss of control compared to PEC, therefore, valine was defined as the main differentiating factor between the studied groups (Tarancon-Diez et al., 2019). That is, elevated valine levels could be a potential biomarker for the prediction of virological progression in controllers and progressors.

***Alterations associated to controllers:*** l-glutamic acid and pyruvate are differentially altered in controllers; these metabolites modulate latent HIV reactivation and/or macrophage inflammation *in vitro* (Giron et al., 2021). A previous study demonstrated that glutamic acid was elevated in Persistent Elite Controllers (PEC) compared to Transient HIV Elite Controllers (TEC) (Tarancon-Diez et al., 2019), suggesting that glutamate metabolism is associated with a delay in the recovery time from HIV.

Likewise, in controllers, acetate is differentially decreased. This metabolite is transiently released into the circulation in response to systemic bacterial infection, as a resistance mechanism of the host’s adaptive immune system. (Bose et al., 2019) (Balmer et al., 2016) (Vysochan et al., 2017). The virus-associated mechanism may be related to the group of HIV controllers, where downregulation of acetate concentration would slow down the lipogenesis.

***Alterations associated to HESN:*** In all groups (controllers, progressors and HESN) evaluated, changes in the lipid profile were observed. However, HESNs showed variations in response compared to controllers and progressors; all significant changes were downward. It highlights the Low-density lipoproteins (LDL) signals that are decreased in HESN and increased in the other groups. LDLs are considered proinflammatory lipid species (Brennan et al., 2021) and associated with immune activation in HIV-infected persons (Funderburg and Mehta, 2016). Other NMR metabolomics studies support our findings on altered lipid metabolism in HIV-infected people (Hewer et al., 2006; Riddler et al., 2008; Philippeos et al., 2009; Swanson et al., 2009; Rodríguez-Gallego et al., 2018; Sitole et al., 2019). We did not identify previous studies that used NMR metabolomics to characterize HESNs as done in this study; however, a previous study that included 32 HESN individuals demonstrated that immune status secondary to HIV exposure influences the plasma efflux capacity of HDL cholesterol, which is buffered in HESN (Tort et al., 2018).

Likewise, glucose was altered in all groups. It is decreased in HESN and increased in the other groups. Early steps of virus replication are moderately affected by the ability of the target cell to perform glycolysis at the time of infection. Similarly, virion production in cultures containing galactose was reduced by 20%–60% compared to the amount produced in glucose-containing cultures (Hegedus et al., 2014). That suggests that high glucose availability in the body is associated with infection process and virus replication, which do not occur in HESN.

The HESN group showed higher lactate expression compared to healthy controls, lactate has anti-inflammatory effects modeling the production of interleukins and other proinflammatory molecules (Hearps et al., 2017) (Aldunate et al., 2013). This suggests a protective role of lactate in the sexual transmission of HIV.

Phosphocholine was found significantly decreased in the HESN group. Phosphocholine shown to be able to suppress immune response in human placenta (Lovell et al., 2007), initiate phagocytic immune recognition (Thompson et al., 1999) and is an intermediate in the synthesis of phosphatidylcholine in tissues (El-Bacha et al., 2016). Phosphatidylcholine has anti-inflammatory effects (Treede et al., 2007/07). The low concentration of phosphocholine in HESN could be related to the production of phosphatidylcholine that would reduce the inflammation of the colon and rectum that occurs in anal intercourse, a common means of HIV exposure in HESN.

It should be noted that cell activation and inflammation have been reported to enhance infection. (Masson et al., 2015; Liebenberg et al., 2017; Wall et al., 1994). The metabolites identified in HESN, and the metabolic and signaling pathways associated with these metabolites, may contribute to the reduction of inflammation and cell activation. Inflammation increases the risk of contracting HIV by causing the activation of HIV target cells (CD4^+^ T cells), increasing their susceptibility to HIV infection (Koning et al., 2005). Inflammation also leads to increased recruitment of these activated target CD4^+^ T cells at the site of HIV exposure (Arnold et al., 2016).

A specific comparison of progressors and HESN revealed that Myo-inositol was elevated in progressors and decreased in HESN. Myo-inositol is a marker of glial reactivity, gliosis and neuroinflammation (Bertran-Cobo et al., 2022). Previously, it was found to be elevated in different brain regions by Magnetic Resonance Spectroscopy (MRS) studies of HIV-infected patients with cognitive impairment (Chang et al., 2004; Cohen et al., 2010; Cassol et al., 2013; Cysique et al., 2013). A previous NMR study on CSF found that impairments in late recall and motor function were associated with higher levels of myo-inositol (Dickens et al., 2015). Alterations in myo-inositol levels were also identified in human mouthwashes (Ghannoum et al., 2013) and brain tissue of HIV-infected rodents (Epstein et al., 2013). All these findings, including those presented in our research, suggest that elevated levels of myo-inositol promote HIV progression in infected people and resistance to infection in HESN.

### HIV-related genes associated with significant metabolites

**FIGURE 7:**
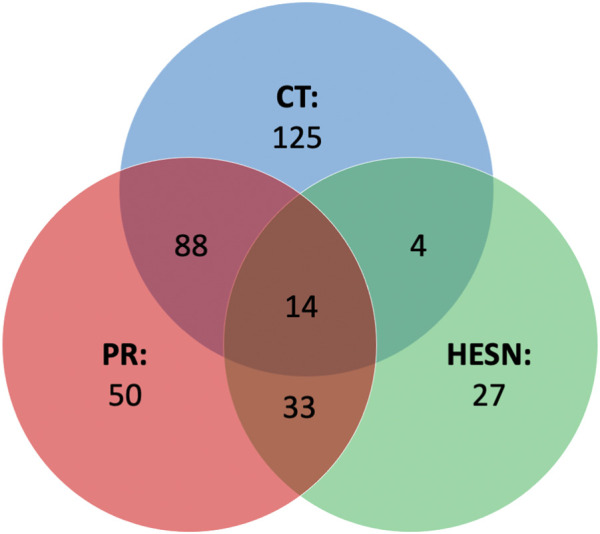
Venn diagram of HIV-related genes. Genes identified by Metabolite-Gene-Disease Interaction Network analysis and filtered through the NCBI gene database as related to HIV-1. In the main part of each set, the genes are related only to one study group and in the intercepts, the genes are related to two or more groups (central intercept). CT: controllers (In blue color set), HESN: HIV-exposed seronegative (In green color set), PR: Progressors (In red color set).

In addition to the altered metabolic pathways, we also wanted to study the genes that were related to these pathways, whose expression could be altered. An analysis of the genes associated to HIV-1 that were related to the metabolites responsible for the difference between the study groups and the healthy controls (See Supplementary Table S6), revealed that patients living with HIV (Controllers and progressors) had the highest number of genes involved in their infectious status (231 and 185 genes *versus* 78 for HESN) (Figure 7). affected by the expression or differential regulation of the metabolites. A total of 341 genes.

We further obtained a more reduce list of genes by filtering only those found in two or more study groups or related to two or more metabolite (Table 1). From these, 21 genes were specifically related to replications interactions (Table 2). The table also summarizes how the alteration of these genes has been previously related to HIV.

**TABLE 1:** HIV-related genes that were associated with two or more study groups and/or metabolites.

Gene	PR	CT	HESN
GLUD2	ALA↓ GLN↑	GLU↓ GLN↑	ALA↓
DNAJB1 PABPN1 FOXP2	ALA↓ GLN↑	GLN↑	ALA↓
LDHA LDHB LDHC	CRE↑ Cre↑ ALA↓	GLU↓ PYR↑	ALA↓ LAC↑
MDH2	ALA↓	GLU↓ PYR↑	ALA↓
PKLR PKM	ALA↓ CRE↑	PYR↑	ALA↓ LAC↑
SLC38A2 SLC38A1	ALA↓ GLN↑ MET↑		ALA↓
GPT2	ALA↓ Cre↑	GLU↓ PYR↑	ALA↓
SLC16A10	ALA↓ GLN↑ MET↑ TYR↓ VAL↑		ALA↓
ARG2	CRE↑ VAL↑	GLU↓ VAL↓	N/A
SERPINC1 CAD CREBBP F13A1 MTOR HTT HIP1 DNAJA1 HSPA1A HSPA4 HSPB1 KCNN3 PML PPP2R2B MAPK8 PSMD2 ATXN2 TAF4 TGM4 TGM1 TGM3 UBA52 SUM O 1 UFD1 VCP VEGFA NCOA3 HAP1 TGM5 HDAC6 DNAJB6 STUB1 BAIAP2 TARDBP UBQLN2 ASRGL1 RBM17 TGM7 TMEM37 TGM6	GLN↑		
BDNF CREB1 GAPDH GART GRIN2B IMPDH2 JUN MAPT MSN PFAS ALDH18A1 QARS1 SPTBN TGM2 NME6	GLN↑	GLU↓ GLN↑	
CASP3	GLN↑ VAL↑	GLU↓ GLN↑ VAL↓	
CAT	CRE↑ MET↑ TYR↓	GLU↓ MET↑	
COMT	VAL↑	GLU↓ VAL↓	
DARS1 EPRS1 GLUL KARS1 RARS1 AIMP2 AIMP1 EEF1E1 LARS1	GLN↑ MET↑	GLU↓ GLN↑ MET↑	
DLD	VAL↑	GLU↓ PYR↑ VAL↓	
F2	Cre↑	GLU↓	
FM O 3 HBB MAT2A MSRA MTR MTRR MYH9 SMUG1	MET↑		
GAD1 SOD1	MET↑	GLU↓ MET↑	
IARS1	GLN↑ MET↑ VAL↑	GLU↓ GLN↑ MET↑ VAL↓	
IGF1 OAT PFKM	CRE↑	GLU↓	
MARS1	Cre↑ GLN↑ MET↑	GLU↓ GLN↑ MET↑	
TH	TYR↓	GLU↓	
MAP3K14	Cre↑ GLN↑	GLN↑	
ALB	Cre↑ TYR↓	N/A	PHO↓
BGLAP	Cre↑		LAC↑
FOXL2 FTL HMOX1 HOXA13 LYZ PIN1 PPIA SRSF1 SLC7A5 KYNU	ALA↓		ALA↓
CD79A CRP	Cre↑		PHO↓
DNMT1 FCN2 GCK B4GALT1 HK2 IFNB1 LGALS3 PYGL SGCB B4GALT2 SIGLEC5 H6PD SIGLEC7 CD207 GBA2 GXYLT1	Glc↑		Glc↓
F3	Cre↑ Glc↑		Glc↓
GYPA	CRE↑ Glc↑		Glc↓
FOS	N/A	GLU↓	LAC↑
OXCT1		3 HB↓	
PLA2G1B		GLU↓	PHO↓
LDHD		PYR↑	LAC↑
ALPI MPO	Cre↑ TYR↓	N/A	N/A
GAA CRNKL1 PIK3C2A	CRE↑ Cre↑		

CT: controllers, HESN: HIV-exposed seronegative, PR: progressors, N/A: Not Applicable. Variation: ↓ (Smaller area or relative concentration in the reference method) ↑ (Bigger area or relative concentration in the reference method). 3-HB: 3-Hydroxybutyrate, ALA: alanine, CRE: creatine, Cre: Creatinine, Glc: d-Glucose, GLN: glutamine, GLU: glutamate, LAC: lactate, MET: methionine, PHO: phosphocholine, PYR: pyruvate, TYR: tyrosine, VAL: valine.

**TABLE 2:** List of genes differentially expressed in the study groups and associated with replication and protein interactions with HIV-1.

Description	Progressors	Controllers	HESN	Replication and/or protein interactions with HIV-1
DnaJ heat shock protein family (Hsp40) member B1	ALA↓ - GLN↑	GLN↑	ALA↓	Knockdown of DnaJ inhibits HIV-1 replication in HeLa-derived TZM-bl cells Brass et al., 2008, while an increase in gene expression is relevant for Tat recruitment in HIV-infected cells Dhamija et al., 2015
				Hsp40 protein is required for HIV-1 Nef-mediated enhancement of viral gene expression and replication Kumar and Mitra, (2005), and that members of this family of interferon-inducible proteins should be considered within its anti-HIV function Urano et al., 2013
Pyruvate kinase L/R	ALA↓ - CRE↑	PYR↑	ALA↓ - LAC↑	*PKLR* has shown a regulatory role in HIV replication in HeLa P4/R5 cells (Zhou et al., 2008)
solute carrier family 38 member 2	ALA↓ - GLN↑ - MET↑	GLU↓ - GLN↑ - MET↑	ALA↓	Down-regulation of *SLC38A1* and *SLC38A2* is associated with HIV interference with immunometabolism in activated primary human CD4^+^ T cells Matheson et al., 2015
solute carrier family 38 member 1	ALA↓ - GLN↑ - MET↑	GLU↓ - GLN↑ - MET↑	ALA↓	This gene uses alanine as an endogenous substrate for T cell mitogenesis Matheson et al., 2015
Glutamic--pyruvic transaminase 2	ALA↓ Cre↑	GLU↓ PYR↑	ALA↓	Knockdown has been shown to inhibit early stages of HIV-1 replication in an *in vitro* model König et al., 2008
Caspase-3	GLN↑ VAL↑	GLU↓ GLN↑ VAL↓	N/A	Is related to HIV-associated dementia (HAD) Yndart et al., 2015 (104)
Coagulation factor II thrombin	Cre↑	GLU↓		Encodes the protein prothrombin Smolkin and Perrotta, (2017). Knockdown of *F2* has previously been suggested to have a regulatory role in HIV replication Zhou et al., 2008
				Thrombin was shown to activate gp120/gp41 of HIV-1, enhances virus-cell fusion Cheng et al., 2010, and enhance the gp160-mediated fusion of HIV-1 with R5 tropism Ling et al., 2004
Glyceraldehyde-3-phosphate dehydrogenase	GLN↑	GLU↓ GLN↑		Negatively regulates HIV-1 infection by directly interacting with *Gag* and *Gag-Pol* Kishimoto et al., 2012
Glutamate ionotropic receptor NMDA type subunit 2B	GLN↑	GLU↓ GLN↑		*GRIN2B* deletion inhibits HIV-1 replication in HeLa P4/R5 cells Zhou et al., 2008, this inhibition is related to HIV-gp120 and Tat upregulating *GRIN2B* Che et al., 2014; Xiong et al., 2014
Lysyl-trna synthetase 1	GLN↑ MET↑	GLU↓ GLN↑ MET↑		Knockdown inhibited the initial stages of HIV-1 replication *in vitro* König et al., 2008
Methionyl-trna synthetase 1	Cre↑ GLN↑ MET↑	GLU↓ GLN↑ MET↑		Knockdown inhibited HIV-1 replication Yeung et al., 2009
Moesin	GLN↑	GLU↓ GLN↑		Knockdown inhibited HIV-1 replication Yeung et al., 2009
Phosphofructokinase muscle	CRE↑	GLU↓		Knockdown inhibited the initial stages of HIV-1 replication *in vitro* König et al., 2008
Mitogen-activated protein kinase 14	Cre↑ GLN↑	GLN↑		MAP kinases (MAPK) have been associated with HIV proteins such as *gp120* Jin et al., 2016, *Nef* Hashimoto et al., 2014, *Tat* Planès et al., 2016 and *Vpr* Hoshino et al., 2010, which generate a strong activation of these enzymes
Aminoacyl trna synthetase complex interacting multifunctional protein 1	GLN↑ MET↑	GLU↓ GLN↑ MET↑		Knockdown inhibited HIV-1 replication Yeung et al., 2009
Leucyl-trna synthetase 1	GLN↑ MET↑	GLU↓ GLN↑ MET↑		Knockdown of DnaJ inhibits HIV-1 replication in HeLa-derived TZM-bl cells Brass et al., 2008
Ficolin 2	Glc↑	N/A	Glc↓	Ficolin-2 binds to HIV-1 *gp120* and blocks viral infection (Luo et al., 2016)
Glucokinase	Glc↑		Glc↓	Knockdown inhibited HIV-1 replication in HeLa-derived TZM-bl cells Brass et al., 2008
Interferon beta 1	Glc↑		Glc↓	Interferon-beta, encoded by *IFNB1* gene, has antiviral, antibacterial and anticancer properties Graber et al., 2014. HIV-1 replication upregulates the expression of *IFNB1* gene Czubala et al., 2016
Galectin 3	Glc↑		Glc↓	In HIV infection, deletion of *LGALS3* by shRNA was shown to inhibit HIV-1 production *in vitro* Wang et al., 2014. Furthermore, it promotes HIV-1 budding through association with Alix and Gag p6 Wang et al., 2014
Sialic acid binding Ig like lectin 5	Glc↑		Glc↓	Siglec-5 associated with divergent outcomes of HIV-1 infection in human and chimpanzee CD4 T cells Soto et al., 2013
Phospholipase A2 group IB	N/A	GLU↓	PHO↓	This gene was involved in CD4 anergy and CD4 lymphopenia in HIV-infected patients Pothlichet et al., 2020
Myeloperoxidase	Cre↑ TYR↓	N/A	N/A	Knockdown of MPO inhibits HIV-1 replication in HeLa P4/R5 cells Zhou et al., 2008
Crooked neck pre-mrna splicing factor 1	CRE↑ Cre↑			CRNKL1 was identified as a highly Selective Regulator of Intron-Retaining HIV-1 and Cellular mRNAs Xiao et al., 2021

CT: controllers, HESN: HIV-exposed seronegative, PR: progressors, N/A: Not Applicable. Variation: ↓ (Smaller area or relative concentration in the reference method) ↑ (Bigger area or relative concentration in the reference method). 3-HB: 3-Hydroxybutyrate, ALA: alanine, CRE: creatine, Cre: Creatinine, Glc: d-Glucose, GLN: glutamine, GLU: glutamate, LAC: lactate, MET: methionine, PHO: phosphocholine, PYR: pyruvate, TYR: tyrosine, VAL: Valine.

Among the genes listed in Table 2, five are related to the three study groups (progressors, controllers and HESN): *DnaJ heat shock protein family (Hsp40) member B1,* Pyruvate kinase liver and red blood cell (*PKLR*) gene, Solute carrier gene family *SLC38A1* and *SLC38A2*, and glutamic-pyruvic transaminase 2 (*GPT2*).

High pyruvate expression in controllers may be associated with elevated PKLR function, whereas in HESN, high lactate expression that is associated with reduced pyruvate would explain a differential gene response in these two groups. *SLC38A1* and *SLC38A2* uses alanine as an endogenous substrate for T cell mitogenesis (Matheson et al., 2015). This metabolite is markedly down regulated in HESN and progressors, but not in controllers, which instead have a low concentration of GLU. In contrast to the HESN, progressors and controllers increase GLN and MET metabolites.

*GPT2* encodes a mitochondrial alanine transaminase, a pyridoxal enzyme that catalyzes the reversible transamination between alanine and 2-oxoglutarate to generate pyruvate and glutamate (Qing et al., 2016). The differential regulations of pyruvate and glutamate in Controllers may be related to a differential expression of this gene and low rates of virus replication.

On the other hand, when the association between HIV progressors and controllers was analyzed, eleven genes (*CASP3* gene, coagulation factor II thrombin gene (*F2*), Glyceraldehyde-3-phosphate dehydrogenase (*GAPDH*), Glutamate ionotropic receptor NMDA type subunit 2B (*GRIN2B*), Mitogen-activated protein kinase 14 (*MAP3K14*) gene, Lysyl-trna synthetase 1 (*KARS*), Phosphofructokinase muscle (*PFKM*), Methionyl-tRNA synthetase 1 (*MARS*) Moesin (*MSN*), Aminoacyl tRNA synthetase complex interacting multifunctional protein 1 (*AIMP1*)) were identified expressed in both groups, but related to different metabolites or opposite metabolite concentration changes (See Table 2).

Between progressors and controllers there are two differences in terms of the associated metabolites: the expression of valine (elevated in progressors and decreased in controllers) and the expression of glutamate that is exclusively decreased in controllers. For this reason, glutamate is then the most important metabolite in the difference between progressors and controllers. Glutamate causes neuronal cell death by apoptosis at high concentrations (Froissard and Duval, 1994; Behl et al., 1995) and glutamate-induced apoptotic cell death was associated with caspase-3 gene regulation (Zhang and Bhavnani, 2006). It could then be stated that low concentrations of glutamate in Controllers may be related to a neuroprotective profile and regulation of apoptosis in HIV infection.

The relationship between prothrombin and/or thrombin with glutamate has been previously determined (Hoogland et al., 2005; Chinnaraj et al., 2018). Thus, the differential expression of glutamate in controllers may be related to an optimized cell proliferation mediated by *F2* that participates in resistance to HIV progression.

There is a relationship between glutamate and *GAPDH* shown previously (Ikemoto et al., 2003), so, it can be inferred that the differential levels observed in controllers can promote the downregulation of the infection.

Creatinine was only found to be altered in progressors. It has been demonstrated that dietary supplementation with creatinine generates a decrease in MAPK expression (Alves et al., 2012); this could occur in progressors with high creatinine levels. MAP kinases (MAPK) are involved in cellular processes such as development, proliferation, differentiation, and transcription regulation (Plotnikov et al., 2011).

Furthermore, in our study we were able to identify five genes that were associated with progressors and HESN: *Ficolin 2* (*FCN2*), *Glucokinase* (*GCK*), *Interferon-beta 1* (*IFNB1*), *Galectin 3* (*LGALS3*), and *Sialic acid binding Ig like lectin 5* (*SIGLEC5*) (See Table 2). All five genes are associated with d-Glucose regulation in study groups. In the progressors, d-Glucose is upregulated and downregulated in the HESNs group. Glucokinase is a type IV isozyme found exclusively in the liver. It is highly specific, only uses d-glucose as a substrate (Sanchez Caballero et al., 2021); it is encoded by the GCK gene, and its knockdown inhibited HIV-1 replication in HeLa-derived TZM-bl cells (Brass et al., 2008). The low concentration of d-Glucose in HESNs may be related to this phenomenon.

It was possible to identify a gene that is related to controllers and HESN, phospholipase A2 group IB gene (*PLA2G1B*). Recently, this gene was involved in CD4 anergy and CD4 lymphopenia in HIV-infected patients (Pothlichet et al., 2020). This gene could potentially be differentially expressed between controllers and HESN. It is enough to identify that HESN present a decreased concentration of PHO, a metabolite directly related to *PLA2G1B*.

Finally, within the replication interactions, two progressor-specific genes associated with increased expression of CRE and Cre were identified: Myeloperoxidase (MPO) and Crooked neck pre-mRNA splicing factor 1 (CRNKL1).

## Conclusion

This study presents differential metabolic profile for controllers, progressors and HESN individuals. Thus, the resistance to HIV-1 progression is associated with changes in the individual’s metabolome, represented in the form of metabolites, that could provide biomarkers of the infectious status of PLHIV, and it will be key to determine the factors that control the infection. Based on our results, we propose tyrosine, glutamate, and valine as biomarkers of progression in HIV infection in progressors and controllers.

It should be noted that the variation in the viral load during progression could affect the metabolomic profile of these individuals. Therefore, conducting a longitudinal study with this population help to resolve this issue. However, according to the HIV “test and treat” guidelines, it is challenging to recruit HIV-positive individuals without receiving ART; therefore, evaluations spanning continuous years of suppressive or not suppressive ART compared with our cohort can help to elucidate the impact of the ART in the metabolomic profile.

Likewise, our study visualized that natural resistance to HIV-1 infection in HESN individuals is associated with a specific metabolic fingerprint, described here for the first time according to our research. We consider LDL, glucose, lactate and Phosphocholine plausible biomarkers of natural resistance to HIV infection in HESN. However, additional studies should be carried out with other HESN groups: female sex workers (FSWs), children born to HIV infected mothers and men who have sex with men (MSM). These analyses allow us to compare and contrast our results, to determine if the metabolites are repeated in the different groups or if there are other phenotypes associated with HIV resistance.

The specific biomarkers for each group were associated with genes and proteins related to HIV-1, therefore, differential expression among groups could potentially explain the characteristics of each group. Finally, we consider that proteomic, transcriptomic and genomic analyzes should be carried out to have a more comprehensive look at the progression and the natural resistance to HIV-1 infection.

## Data Availability

The original contributions presented in the study are included in the article/Supplementary Material, further inquiries can be directed to the corresponding authors.
