# Supplementary figures and images for: Plasma metabolomics by nuclear magnetic resonance reveals biomarkers and metabolic pathways associated with the control of HIV-1 infection/progression
Source: Front Mol Biosci. 2023 Jun 29;10:1204273. doi: 10.3389/fmolb.2023.1204273 (PMC10339029; doi:10.3389/fmolb.2023.1204273)

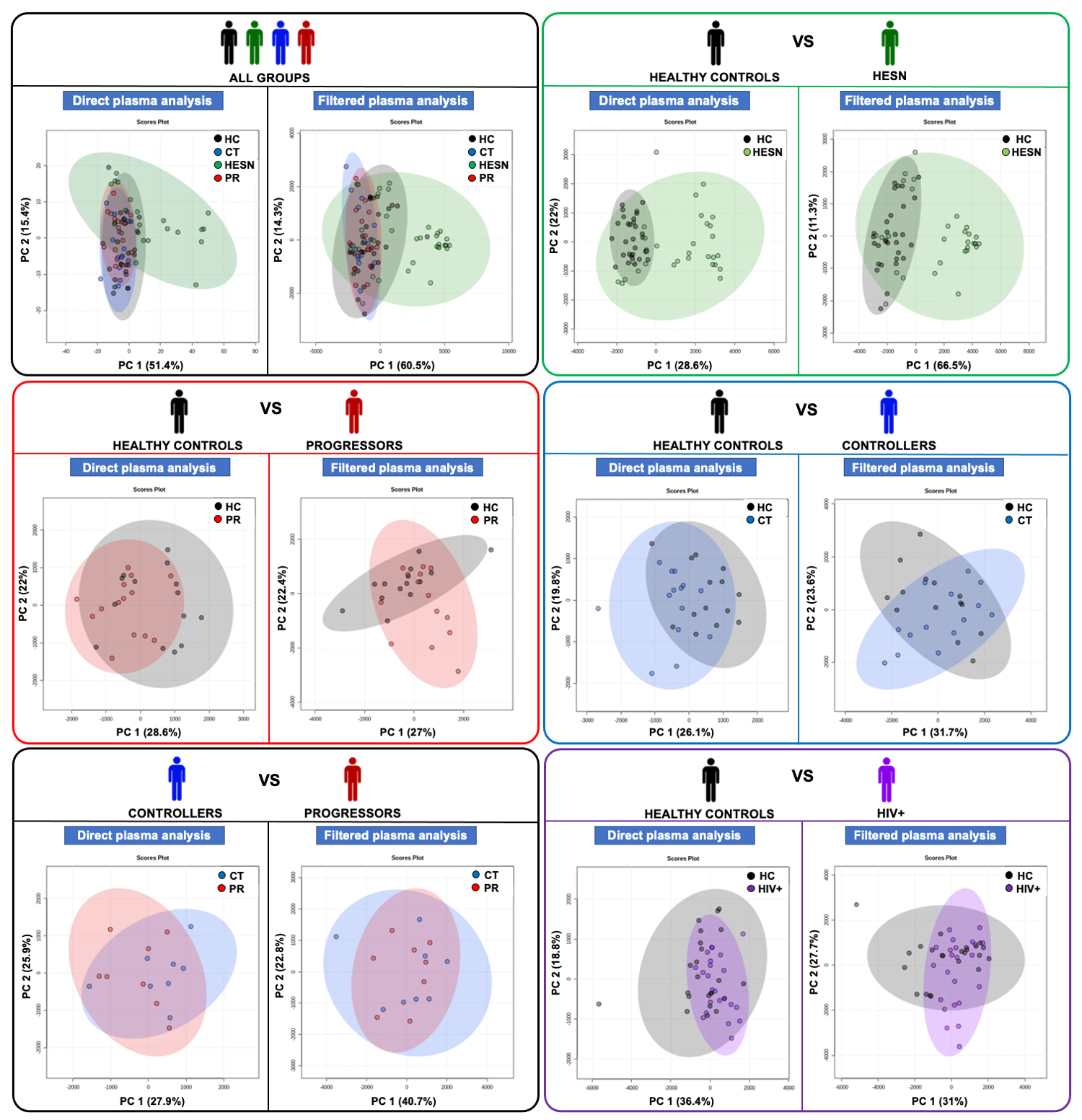

Supplement: Supplementary file 1 [file Image3.TIFF]

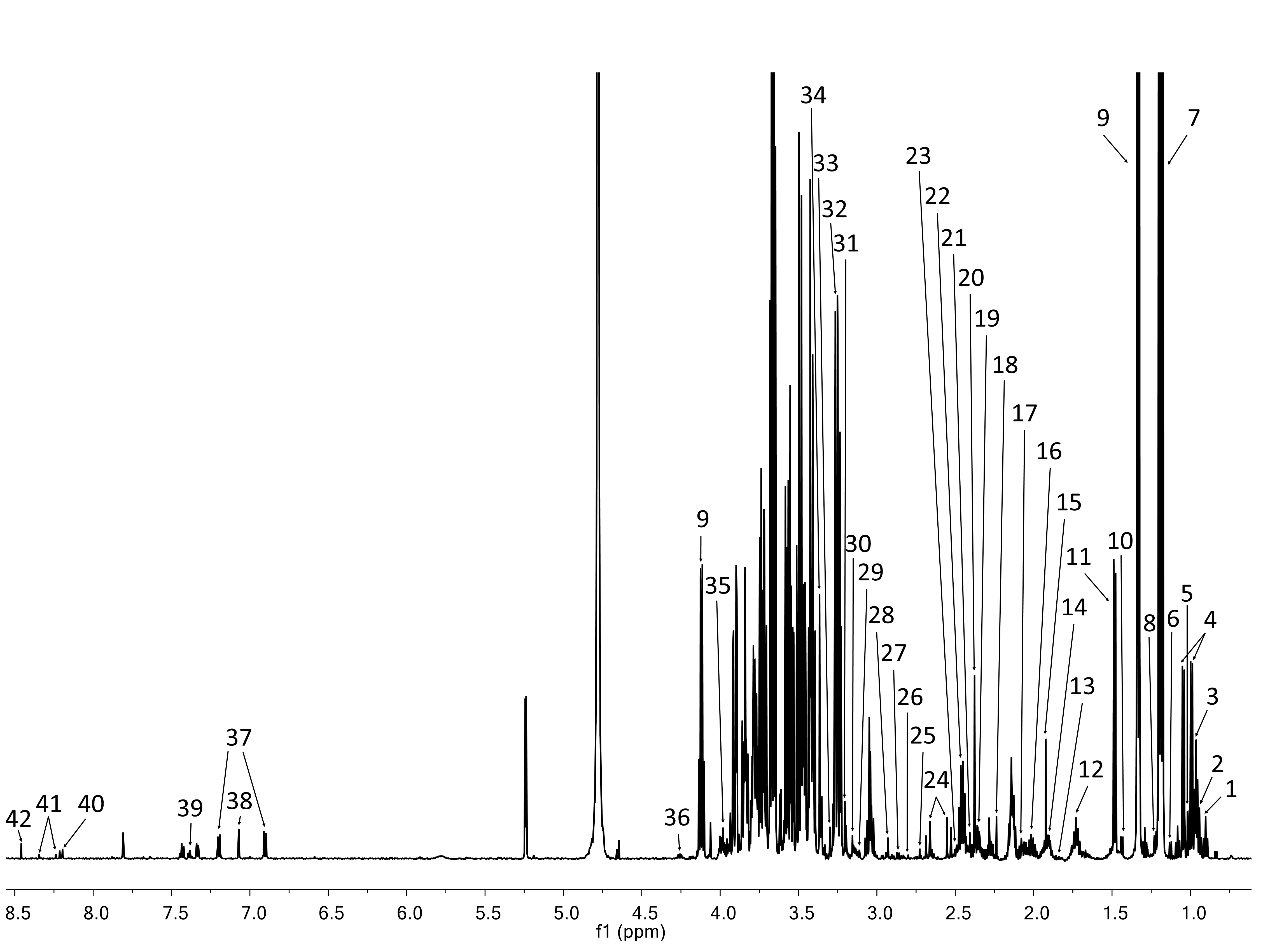

Supplement: Supplementary file 5 [file Image2.TIF]

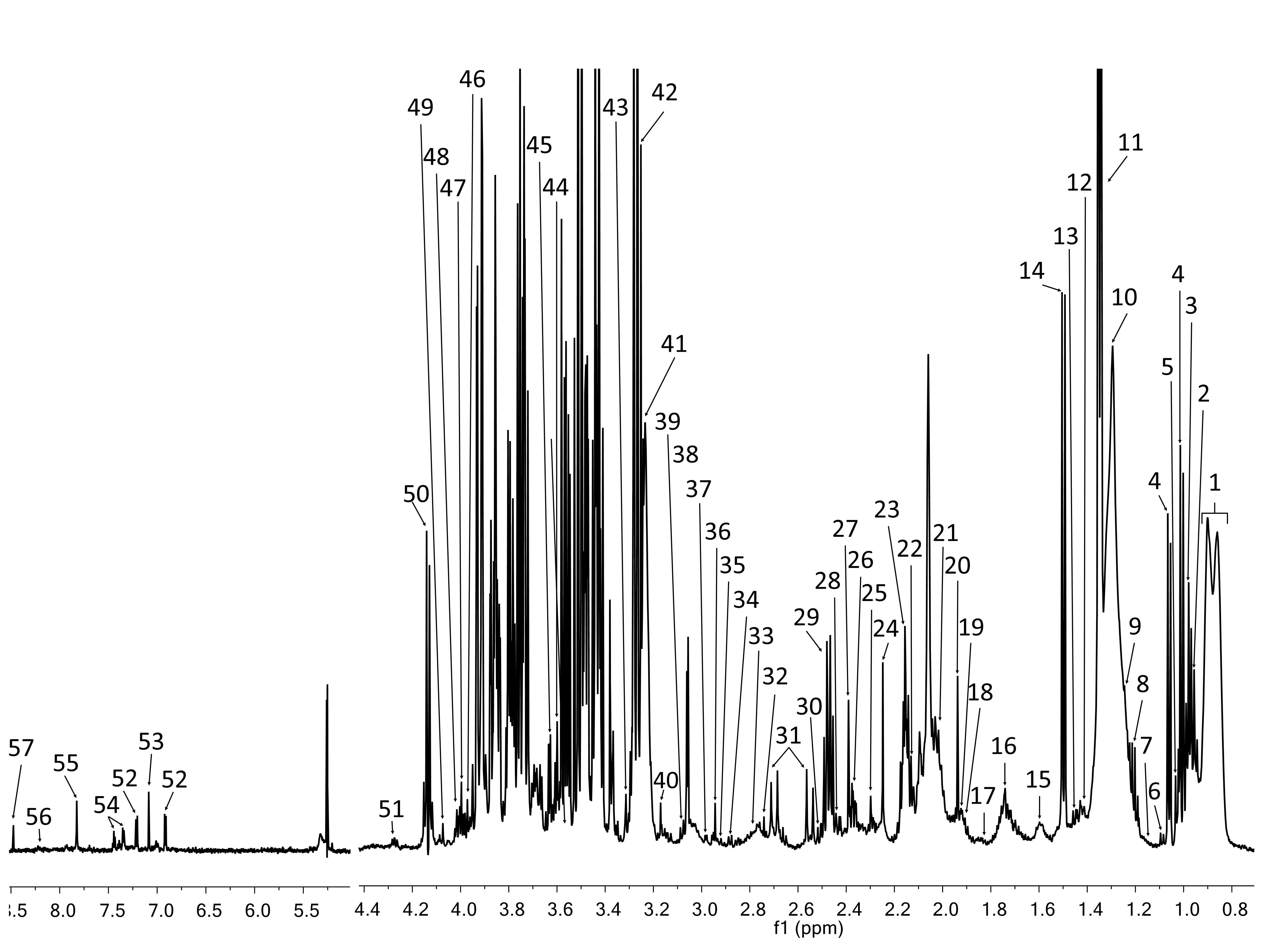

Supplement: Supplementary file 6 [file Image1.TIF]

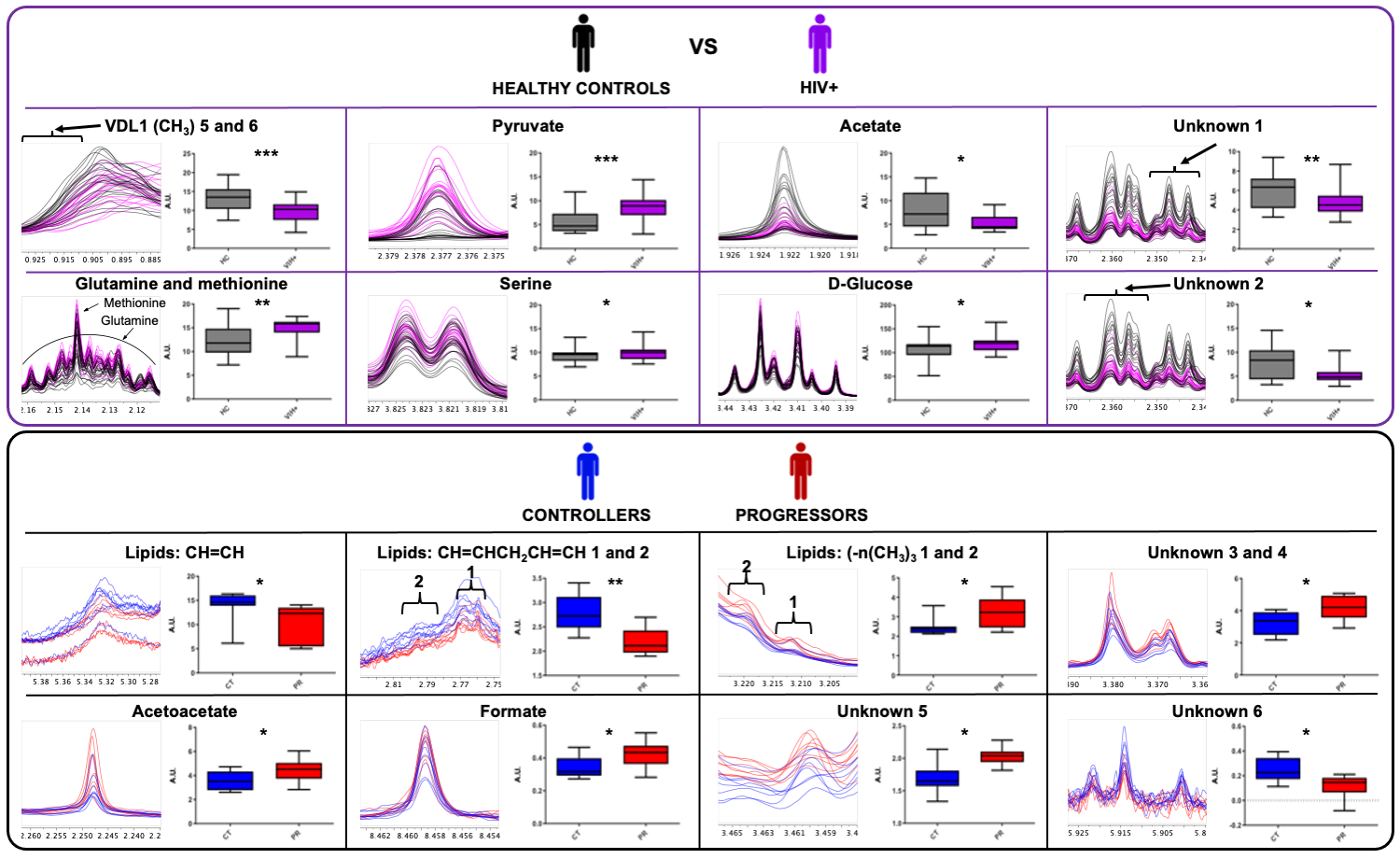

Supplement: Supplementary file 11 [file Image4.TIFF]
