# Supplementary material for: Plasma metabolomics by nuclear magnetic resonance reveals biomarkers and metabolic pathways associated with the control of HIV-1 infection/progression
Source: Front Mol Biosci. 2023 Jun 29;10:1204273. doi: 10.3389/fmolb.2023.1204273 (PMC10339029; doi:10.3389/fmolb.2023.1204273)
Supplement: Supplementary file 2 [file DataSheet2.PDF]

Names	total	elements
CT HESN PR	14	PKM2
		FOXP2
		SLC38A1
		DNAJB1
		LDHA
		SLC38A2
		GLUD2
		GPT2
		PKLR
		MDH2
		LDHB
		LDHC
		SLC16A10
		PABPN1
CT PR	88	MSRA
		MTOR
		TGM1
		HSPB1
		STUB1
		QARS
		NCOA3
		ARG2
		IGF1
		NME6
		SUMO1
		TH
		PFAS
		PML
		CREBBP
		DLD
		MSN
		CAT
		F13A1
		PPP2R2B
		TGM4
		NMNAT1
		DARS
		TGM2
		EPRS
		GRIN2B
		TGM7
		IMPDH2
		F2
		DNAJA1
		RBM17
		FMO3
		MTRR
		TGM3
		MARS
		SOD1
		RARS
		SMUG1
		ALDH18A1

	PSMD2
	HTT
	ATXN2
	CREB1
	HSPA1A
	KCNN3
	BDNF
	TGM6
	SERPINC1
	CAD
	MAP3K14
	ASRGL1
	MYH9
	TAF4
	GAPDH
	EEF1E1
	GLUL
	IARS
	PFKM
	VEGFA
	HIP1
	GART
	LARS
	AIMP1
	MAPK8
	UBA52
	DNAJB6
	JUN
	HAP1
	UFD1L
	GAD1
	VCP
	AIMP2
	HSPA4
	BAIAP2
	SPTBN2
	MAT2A
	MAPT
	TMEM37
	TARDBP
	KARS
	COMT
	HBB
	MTR
	HDAC6
	CASP3
	TGM5
	UBQLN2
	OAT
CT HESN 4	OXCT1
	LDHD
	PLA2G1B
	FOS
HESN PR 33	HMOX1

		SIGLEC5
		GXYLT1
		BGLAP
		HOXA13
		LYZ
		CD207
		B4GALT2
		KYNU
		PYGL
		DNMT1
		SLC7A5
		CD79A
		LGALS1
		GYPA
		PIN1
		LGALS3
		ALB
		F3
		FOXL2
		H6PD
		PPIA
		IFNB1
		SRSF1
		SIGLEC7
		HK2
		GCK
		B4GALT1
		SGCB
		CRP
		GBA2
		FTL
		FCN2
CT	125	MMP2
		FH
		ENO1
		PDHA1
		TKT
		ENG
		GRM7
		AIFM1
		TGFB2
		SLC1A3
		TP53
		CYCS
		GRM3
		EEF2
		SNAP25
		PRKCG
		NOS2
		RBFOX3
		CAMK2B
		DLG4
		GLRB
		PLCB2

NGFR
NLRP1
CASP9
GRIN3B
UROD
FGFR1
SDHB
CDK5
CNR2
NGF
SLC1A2
NTF4
GSTZ1
LEP
CAMK1
CSF3
TGFB3
ATP5O
AKT1
CDO1
GSTM1
NOS1
PRL
PLCB1
ADCY2
PHGDH
FPGS
GRIA1
HSD17B12
OGDH
GSS
SLC16A6
SLC25A12
BIRC5
HMOX2
SLC1A7
GRM4
NFKB1
CD38
NT5E
PDC
RASGRF1
S100B
TAC1
SST
SLCO2A1
GRIN2D
CDKN1B
PSAT1
GRIN1
SORT1
GCLM
BCL2
CAMK2A

		KL
		HRAS
		GRM5
		PRODH
		RAB3A
		IDH2
		AKAP9
		ADCY8
		SLC7A11
		ADORA2A
		SYN1
		GRIN2A
		PVALB
		PDHB
		GRIA3
		BCL2L1
		TACR1
		PLCB3
		PENK
		LIF
		CALM1
		ACHE
		BAX
		GRIN2C
		SIGMAR1
		RHOA
		AMD1
		GSTT1
		GGT5
		GCLC
		MAP2
		NOS3
		GSTP1
		IL6
		CAMK2G
		REPIN1
		APP
		GRIA2
		EGR1
		SLC2A1
		ABCB1
		PPP3CC
		GRIN3A
		GRINA
		SYP
		RNF213
		NR3C1
		EEF1A2
		GFAP
PR	50	ACE
		ACTN4
		C4B
		EDN1
		GSR

		PRKAA2
		CRNKL1
		YARS
		CIT
		DYRK1A
		CKMT1B
		PPARA
		MPO
		TTR
		SLC22A2
		EDNRB
		SPP1
		FABP1
		EGFR
		CYGB
		SLC25A4
		NPPB
		REN
		LCN2
		KAT8
		CEACAM1
		SNRNP40
		NKRF
		GPR182
		PPBP
		HAVCR1
		CFH
		AGT
		EPO
		CST3
		PDXP
		COL1A2
		CCL2
		SLC2A4
		NPHS1
		LRSAM1
		RB1
		ALPI
		YWHAB
		THBD
		YWHAZ
		PIK3C2A
		MRS2
		HBA1
		GAA
HESN	27	PTAFR
		SMPD1
		RPLP0
		PLCH1
		RPL37A
		HDAC4
		CSTB
		POSTN
		RPL11

NHP2L1
RPL8
DYNC1LI1
PLA2G2A
BMP2
DDIT3
C17orf48
ACPP
ATR
DYNC1LI2
SMPD3
PCSK7
SGMS2
CLTC
RPL9
PDE4A
SMPD2
BSG
