# Supplementary material for: Plasma metabolomics by nuclear magnetic resonance reveals biomarkers and metabolic pathways associated with the control of HIV-1 infection/progression
Source: Front Mol Biosci. 2023 Jun 29;10:1204273. doi: 10.3389/fmolb.2023.1204273 (PMC10339029; doi:10.3389/fmolb.2023.1204273)
Supplement: Supplementary file 7 [file DataSheet1.PDF]

GeneID	Symbol	Aliases	description
43	ACHE	ACEE, ARACHE, N-ACHE, YT	acetylcholinesterase (Cartwright blood group)
55	ACP3	5'-NT, ACP-3, ACPP, TM-PAP	acid phosphatase 3
81	ACTN4	ACTININ-4, FSGS, FSGS1	actinin alpha 4
108	ADCY2	AC2, HBAC2	adenylate cyclase 2
114	ADCY8	AC8, ADCY3, HBAC1	adenylate cyclase 8
135	ADORA2A	A2aR, ADORA2, RDC8	adenosine A2a receptor
183	AGT	ANHU, SERPINA8, hFLT1	angiotensinogen
207	AKT1	RAC-ALPHA	AKT serine/threonine kinase 1
213	ALB	PRO1341	albumin
248	ALPI	IAP	alkaline phosphatase, intestinal
262	AMD1	ADOMETDC, AMD, SAMDC	adenosylmethionine decarboxylase 1
291	SLC25A4	AAC1, ANT, ANT 1, ANT1, MTDPS12, MTDPS12A, PEO2, PEO3, PEOA2, T1	solute carrier family 25 member 4
332	BIRC5	API4, EPR-1	baculoviral IAP repeat containing 5
351	APP	CTFgamma, CVAP, PN-II, PN2, alpha-sAPP, preA4, APP	amyloid beta precursor protein
384	ARG2		arginase 2
387	RHOA	RHOH12	ras homolog family member A
462	SERPINC1	AT3, AT3D, ATIII, ATIII-R2, ATIII-T1, ATIII-T2, THPH7	serpin family C member 1
539	ATP5PO	ATP5O, ATPO, HMC08D05, OSCP	ATP synthase peripheral stalk subunit OSCP
545	ATR	FCTCS, FRP1, MEC1, SCKL, SCKL1	ATR serine/threonine kinase
581	BAX	BCL2L4	BCL2 associated X, apoptosis regulator
596	BCL2	Bcl-2, PPP1R50	BCL2 apoptosis regulator
598	BCL2L1	PPP1R52	BCL2 like 1
627	BDNF	ANON2, BULN2	brain derived neurotrophic factor
632	BGLAP	BGP, OC, OCN	bone gamma-carboxyglutamate protein
634	CEACAM1	BGP, BGP1, BGPI	CEA cell adhesion molecule 1
650	BMP2	BDA2A, SSFSC, SSFSC1, BMP2	bone morphogenetic protein 2
668	FOXL2	BPES, BPES1, PFRK, PINTO, POF3	forkhead box L2
682	BSG	5F7, CD147, EMMPRIN, EMPRIN, HAb18G, OK, SLC7A11, TCSF	basigin (Ok blood group)
721	C4B	C4B112, C4B2, C4B3, C4B5, C4BD, C4B_2, C4F, CH, CO4, CPAMD3, C4B	complement C4B (Chido blood group)
790	CAD	CDG1Z, DEE50, EIEE50, GATD4	carbamoyl-phosphate synthetase 2, aspartate transcarbamylase, and dihydroorotase
801	CALM1	CAMI, CAMIII, CPVT4, DD132, LQT14, PHKD, caM	calmodulin 1
815	CAMK2A	CAMKA, CaMKIINalpha, CaMKIIalpha, MRD53, MRT63	calcium/calmodulin dependent protein kinase II alpha

GeneID	Symbol	Aliases	description
816	CAMK2B	CAM2, CAMK2, CAMKB, CaMKIIbeta, MRD54	calcium/calmodulin dependent protein kinase II beta
818	CAMK2G	CAMK, CAMK-II, CAMKG, MRD59	calcium/calmodulin dependent protein kinase II gamma
836	CASP3	CPP32, CPP32B, SCA-1	caspase 3
842	CASP9	PPP1R56	caspase 9
847	CAT		catalase
952	CD38	ADPRC 1, ADPRC1	CD38 molecule
973	CD79A	IGA, MB-1	CD79a molecule
1020	CDK5	LIS7, PSSALRE	cyclin dependent kinase 5
1027	CDKN1B	CDKN4, KIP1, MEN1B, MEN4, P27KIP1	cyclin dependent kinase inhibitor 1B
1036	CDO1	CDO-I	cysteine dioxygenase type 1
1159	CKMT1B	CKMT, CKMT1, UMTCK	creatine kinase, mitochondrial 1B
1213	CLTC	CLTC	clathrin heavy chain
1269	CNR2	CB-2, CB2, CX5	cannabinoid receptor 2
1278	COL1A2	EDSARTH2, EDSCV, OI4	collagen type I alpha 2 chain
1312	COMT	HEL-S-98n	catechol-O-methyltransferase
1385	CREB1	CREB, CREB-1	cAMP responsive element binding protein 1
1387	CREBBP	CBP, KAT3A, MKHK1, RSTS, RSTS1	CREB binding protein
1401	CRP	PTX1	C-reactive protein
1440	CSF3	C17orf33OS, GCSF, CSF3	colony stimulating factor 3
1471	CST3	ARMD11, HEL-S-2	cystatin C
1476	CSTB	STFB, ULD	cystatin B
1615	DARS1	DARS, HBSL, aspRS	aspartyl-tRNA synthetase 1
1636	ACE	ACE1, CD143, DCP, DCP1	angiotensin I converting enzyme
1649	DDIT3	AltDDIT3, C/EBPzeta, CEBPZ, CHOP, CHOP-10, CHOP10, GADD153	DNA damage inducible transcript 3
1738	DLD	PHE3, DLD	dihydrolipoamide dehydrogenase
1742	DLG4	MRD62, PSD95, SAP-90, SAP90	discs large MAGUK scaffold protein 4
1783	DYNC1LI2	DNCLI2, LIC2	dynein cytoplasmic 1 light intermediate chain 2
1786	DNMT1	ADCADN, AIM, CXXC9, DNMT, HSN1E, MCMT, m.HsaI	DNA methyltransferase 1
1859	DYRK1A	DYRK, DYRK1, HP86, MNB, MNBH, MRD7	dual specificity tyrosine phosphorylation regulated kinase 1A
1906	EDN1	ARCND3, ET1, HDLCQ7, PPET1, QME	endothelin 1
1910	EDNRB	ABCDS, ET-B, ET-BR, ETB, ETB1, ETBR, ETRB, HSCR, HSCR2, WS4A	endothelin receptor type B
1917	EEF1A2	DEE33, EEF1AL, EF-1-alpha-2, EF1A, EIEE33, HS1, MRD38, STN, STNL	eukaryotic translation elongation factor 1 alpha 2
1938	EEF2	EEF-2, EF-2, EF2, SCA26	eukaryotic translation elongation factor 2

GeneID	Symbol	Aliases	description
1956	EGFR	ERBB, ERBB1, ERRP, HER1, NISBD2, PIG61, mENA	epidermal growth factor receptor
1958	EGR1	AT225, G0S30, KROX-24, NGFI-A, TIS8, ZIF-268, ZNF225	early growth response 1
2022	ENG	END, HHT1, ORW1	endoglin
2023	ENO1	ENO1L1, HEL-S-17, MPB1, NNE, PPH	enolase 1
2056	EPO	DBAL, ECYT5, EP, MVCD2	erythropoietin
2058	EPRS1	EARS, EPRS, GLUPRORS, HLD15, PARS, PIG32, QARS, QPRS	glutamyl-prolyl-tRNA synthetase 1
2147	F2	PT, RPRGL2, THPH1	coagulation factor II, thrombin
2152	F3	CD142, TF, TFA	coagulation factor III, tissue factor
2162	F13A1	F13A	coagulation factor XIII A chain
2168	FABP1	FABPL, L-FABP	fatty acid binding protein 1
2220	FCN2	EBP-37, FCNL, P35, ficolin-2	ficolin 2
2260	FGFR1	FGFR-1, FLG, FLT-2, FLT2, HBGFR, HH2, HRTFDS, KAL2, N-SAM, OGD, bFGF-R-1	fibroblast growth factor receptor 1
2271	FH	MCUL1	fumarate hydratase
2328	FMO3	FMOII, TMAU, dJ127D3.1	flavin containing dimethylaniline monoxygenase 3
2353	FOS	AP-1, C-FOS, p55	Fos proto-oncogene, AP-1 transcription factor subunit
2356	FPGS		folylpolyglutamate synthase
2475	MTOR	SKS	mechanistic target of rapamycin kinase
2512	FTL	LFTD, NBIA3	ferritin light chain
2548	GAA	LYAG	alpha glucosidase
2571	GAD1	CPSQ1, DEE89, GAD, SCP	glutamate decarboxylase 1
2597	GAPDH	G3PD, GAPD, HEL-S-162eP	glyceraldehyde-3-phosphate dehydrogenase
2618	GART	AIRS, GARSF, PAIS, PGFT, PRGS, GART	phosphoribosylglycinamide formyltransferase, phosphoribosylglycinamide synthetase, phosphoribosylaminoimidazole synthetase
2645	GCK	FGQTL3, GK, GLK, HHF3, HK4, HKIV, HXKP, LGLK, MODY2, PNDM1	glucokinase
2670	GFAP	ALXDRD	glial fibrillary acidic protein
2683	B4GALT1	B4GAL-T1, CDG2D, GGTB2, GT1, GTB, beta4Gal-T1	beta-1,4-galactosyltransferase 1
2687	GGT5	GGL, GGT 5, GGT-REL, GGTLA1	gamma-glutamyltransferase 5
2729	GCLC	GCL, GCS, GLCL, GLCLC	glutamate-cysteine ligase catalytic subunit
2730	GCLM	GLCLR	glutamate-cysteine ligase modifier subunit
2743	GLRB	HKPX2	glycine receptor beta
2747	GLUD2	GDH2, GLUDP1	glutamate dehydrogenase 2

GeneID	Symbol	Aliases	description
2752	GLUL	GLNS, GS, PIG43, PIG59	glutamate-ammonia ligase
2890	GRIA1	GLUH1, GLUR1, GLURA, GluA1, HBGR1	glutamate ionotropic receptor AMPA type subunit 1
2891	GRIA2	GLUR2, GLURB, GluA2, GluR-K2, HBGR2, NEDLIB, gluR-2, gluR-B	glutamate ionotropic receptor AMPA type subunit 2
2892	GRIA3	GLUR-C, GLUR-K3, GLUR3, GLURC, GluA3, MRX94, MRXSW	glutamate ionotropic receptor AMPA type subunit 3
2902	GRIN1	GluN1, MRD8, NDHMSD, NDHMSR, NMD-R1, NMDA1, NMDAR1, NR1	glutamate ionotropic receptor NMDA type subunit 1
2903	GRIN2A	EPND, FESD, GluN2A, LKS, NMDAR2A, NR2A	glutamate ionotropic receptor NMDA type subunit 2A
2904	GRIN2B	DEE27, EIEE27, GluN2B, MRD6, NMDAR2B, NR2B, NR3, hNR3	glutamate ionotropic receptor NMDA type subunit 2B
2905	GRIN2C	GluN2C, NMDAR2C, NR2C	glutamate ionotropic receptor NMDA type subunit 2C
2906	GRIN2D	DEE46, EB11, EIEE46, GluN2D, NMDAR2D, NR2D	glutamate ionotropic receptor NMDA type subunit 2D
2907	GRINA	HNRGW, LFG1, NMDARA1, TMBIM3	glutamate ionotropic receptor NMDA type subunit associated protein 1
2908	NR3C1	GCCR, GCR, GCRST, GR, GRL	nuclear receptor subfamily 3 group C member 1
2913	GRM3	GLUR3, GPRC1C, MGLUR3, mGlu3	glutamate metabotropic receptor 3
2914	GRM4	GPRC1D, MGLUR4, mGlu4	glutamate metabotropic receptor 4
2915	GRM5	GPRC1E, MGLUR5, PPP1R86, mGlu5	glutamate metabotropic receptor 5
2917	GRM7	GLUR7, GPRC1G, MGLU7, MGLUR7, NEDSHBA, PPP1R87	glutamate metabotropic receptor 7
2936	GSR	GRD, HEL-75, HEL-S-122m, GSR	glutathione-disulfide reductase
2937	GSS	GSHS, HEL-S-64p, HEL-S-88n	glutathione synthetase
2944	GSTM1	GTH4, GTM1, H-B, MU, MU-1, GSTM1	glutathione S-transferase mu 1
2950	GSTP1	22, PI	glutathione S-transferase pi 1
2952	GSTT1		glutathione S-transferase theta 1
2954	GSTZ1	GSTZ1-1, MAAI, MAAID, MAI	glutathione S-transferase zeta 1
2993	GYPA	HGpMiV, HGpMiXI, HGpSta(C), MN, MNS, PAS-2	glycophorin A (MNS blood group)
3039	HBA1	ECYT7, HBA-T3, HBH, METHBA	hemoglobin subunit alpha 1
3043	HBB	CD113t-C, ECYT6, beta-globin	hemoglobin subunit beta
3064	HTT	HD, IT15, LOMARS	huntingtin
3075	CFH	AHUS1, AMBP1, ARMD4, ARMS1L3, FH, FHL1, HF, HF1, HF2, HUS, CFH	complement factor H
3092	HIP1	SHONgamma	huntingtin interacting protein 1
3099	HK2	HKII, HXK2	hexokinase 2

GeneID	Symbol	Aliases	description
3162	HMOX1	HMOX1D, HO-1, HSP32, bK286B10	heme oxygenase 1
3163	HMOX2	HO-2	heme oxygenase 2
3209	HOXA13	HOX1, HOX1J	homeobox A13
3265	HRAS	CTLO, H-RASIDX, HAMSV1, RASH1, p21ras, HRAS	HRas proto-oncogene, GTPase
3301	DNAJA1	DJ-2, DjA1, HDJ2, HSDJ, HSJ-2, HSJ2, HSPF4, NEDD7, hDJ-2	DnaJ heat shock protein family (Hsp40) member A1
3303	HSPA1A	HSP70-2, HSP70.1, HSP70.2, HSP70I, HSP72, HSPA1	heat shock protein family A (Hsp70) member 1A
3308	HSPA4	APG-2, HEL-S-5a, HS24/P52, HSPH2, RY, hsp70, hsp70RY	heat shock protein family A (Hsp70) member 4
3315	HSPB1	HS.76067, HSP27, HSP28, Hsp25, SRP27	heat shock protein family B (small) member 1
3337	DNAJB1	HSPF1, Hdj1, Hsp40, RSPH16B, Sis1	DnaJ heat shock protein family (Hsp40) member B1
3376	IARS1	PRO0785	isoleucyl-tRNA synthetase 1
3418	IDH2	D2HGA2, ICD-M, IDH, IDHM, IDP, IDPM, mNADP-IDH	isocitrate dehydrogenase (NADP(+)) 2
3456	IFNB1	IFB, IFF, IFN-beta, IFNB	interferon beta 1
3479	IGF1	IGF, IGF-I, IGFI, MGF	insulin like growth factor 1
3569	IL6	BSF-2, BSF2, CDF, HGF, HSF, IFN-beta-2, IFNB2, IL-6	interleukin 6
3615	IMPDH2	IMPD2, IMPDH-II	inosine monophosphate dehydrogenase 2
3725	JUN	AP-1, AP1, c-Jun, cJUN, p39	Jun proto-oncogene, AP-1 transcription factor subunit
3735	KARS1	CMTRIB, DEAPLE, DFNB89, KARS, KARS2, KRS, LEPID	lysyl-tRNA synthetase 1
3782	KCNN3	KCa2.3, SK3, SKCA3, ZLS3, hSK3	potassium calcium-activated channel subfamily N member 3
3934	LCN2	24p3, MSFI, NGAL, p25	lipocalin 2
3939	LDHA	GSD11, HEL-S-133P, LDHM, PIG19	lactate dehydrogenase A
3945	LDHB	LDHB	lactate dehydrogenase B
3948	LDHC	CT32, LDH3, LDHX	lactate dehydrogenase C
3952	LEP	LEPD, OB, OBS	leptin
3956	LGALS1	GAL1, GBP	galectin 1
3958	LGALS3	LGALS2, MAC2	galectin 3
3976	LIF	CDF, DIA, HILDA, MLPLI	LIF interleukin 6 family cytokine
4069	LYZ	LYZF1, LZM	lysozyme
4133	MAP2	MAP-2A, MAP2B, MAP2C, MAP2	microtubule associated protein 2
4137	MAPT	MTBT2, PPND, PPP1R103, TAU, tau-40, MAPT	microtubule associated protein tau

GeneID	Symbol	Aliases	description
4141	MARS1	CMT2U, ILFS2, ILLD, MARS, METRS, MRS, MTRNS, SPG70	methionyl-tRNA synthetase 1
4144	MAT2A	MATA2, MATII, SAMS2	methionine adenosyltransferase 2A
4191	MDH2	MGC:3559, MOR1	malate dehydrogenase 2
4313	MMP2	MONA, TBE-1	matrix metallopeptidase 2
4353	MPO		myeloperoxidase
4478	MSN	HEL70, IMD50	moesin
4482	MSRA	PMSR	methionine sulfoxide reductase A
4548	MTR	HMAG, MS, cblG	5-methyltetrahydrofolate-homocysteine methyltransferase
4552	MTRR	MSR, cblE	5-methyltetrahydrofolate-homocysteine methyltransferase reductase
4627	MYH9	MATINS, MHA, NMHC-II-A, NMMHC-IIA, NMMHCA	myosin heavy chain 9
4790	NFKB1	NF-kappa-B1, NF-kappaB, NF-kappabeta, NFKB-p105, NFKB-p50, NFkappaB	nuclear factor kappa B subunit 1
4803	NGF	Beta-NGF, HSAN5B, NGF	nerve growth factor
4804	NGFR	CD271, Gp80-LNGFR, TNFRSF16, p75(NTR), p75NTR	nerve growth factor receptor
4809	SNU13	15.5K, FA-1, FA1, NHP2L1, NHPX, OTK27, SNRNP15-5, SPAG12, SSFA1	small nuclear ribonucleoprotein 13
4842	NOS1	nNOS	nitric oxide synthase 1
4843	NOS2	HEP-NOS, INOS, NOSA, NOS2	nitric oxide synthase 2
4846	NOS3	ECNOS, eNOS	nitric oxide synthase 3
4868	NPHS1	CNF, NPHN, nephrin	NPHS1 adhesion molecule, nephrin
4879	NPPB	BNP, Iso-ANP	natriuretic peptide B
4907	NT5E	eN, eNT	5'-nucleotidase ecto
4909	NTF4	GLC10, GLC1O, NT-4, NT-4/5, NT-5, NT4, NT5, NTF5	neurotrophin 4
4942	OAT	GACR, HOGAASE, OKT, OAT	ornithine aminotransferase
4967	OGDH	AKGDH, E1k, KGD1, OGDC2, OGDH	oxoglutarate dehydrogenase
5019	OXCT1	OXCT, SCOT	3-oxoacid CoA-transferase 1
5132	PDC	MEKA, PHD, PhLOP, PhLP	phosducin
5141	PDE4A	DPDE2, PDE4, PDE46	phosphodiesterase 4A
5160	PDHA1	PDHA, PDHAD, PDHCE1A, PHE1A	pyruvate dehydrogenase E1 subunit alpha 1
5162	PDHB	PDHBD, PDHE1-B, PDHE1B, PHE1B	pyruvate dehydrogenase E1 subunit beta
5179	PENK	PE-A, PENK	proenkephalin
5198	PFAS	PURL	phosphoribosylformylglycinamidine synthase
5213	PFKM	ATP-PFK, GSD7, PFK-1, PFK-A, PFK1, PFKA, PFKX, PPP1R122	phosphofructokinase, muscle

GeneID	Symbol	Aliases	description
5243	ABCB1	ABC20, CD243, CLCS, GP170, MDR1, P-GP, PGY1, p-170	ATP binding cassette subfamily B member 1
5286	PIK3C2A	CPK, OCSKD, PI3-K-C2(ALPHA), PI3-K-C2A, PI3K-C2-alpha, PI3K-C2alpha	phosphatidylinositol-4-phosphate 3-kinase catalytic subunit type 2 alpha
5300	PIN1	DOD, UBL5	peptidylprolyl cis/trans isomerase, NIMA-interacting 1
5313	PKLR	PK1, PKL, PKRL, RPK	pyruvate kinase L/R
5315	PKM	CTHBP, HEL-S-30, OIP3, PK32, TCB, THBP1, p58, PKM	pyruvate kinase M1/2
5319	PLA2G1B	PLA2, PLA2A, PPLA2	phospholipase A2 group IB
5320	PLA2G2A	MOM1, PLA2, PLA2B, PLA2L, PLA2S, PLAS1, sPLA2	phospholipase A2 group IIA
5330	PLCB2	PLC-beta-2	phospholipase C beta 2
5331	PLCB3	SMDCD	phospholipase C beta 3
5371	PML	MYL, PP8675, RNF71, TRIM19	PML nuclear body scaffold
5465	PPARA	PPARalpha, hPPAR	peroxisome proliferator activated receptor alpha
5473	PPBP	CTAPIII, CXCL7, LA-PF4, LDGF, MDGF, NAP-2, PBP, SCYB7, TC1, TC2, TGB, TGB1, THBGB, THBGB1	pro-platelet basic protein
5478	PPIA	CYPA, CYPH, HEL-S-69p	peptidylprolyl isomerase A
5521	PPP2R2B	[illegible] PP2ABBETA, PP2APR55B, PP2APR55BETA, PR2AB55BETA, PR2ABBETA, PR2APR55BETA, PR52B, PR55-BETA, PR55BETA, SCA12	protein phosphatase 2 regulatory subunit Bbeta
5533	PPP3CC	CALNA3, CNA3, PP2Bgamma	protein phosphatase 3 catalytic subunit gamma
5563	PRKAA2	AMPK, AMPK2, AMPKa2, PRKAA	protein kinase AMP-activated catalytic subunit alpha 2
5582	PRKCG	PKC-gamma, PKCC, PKCG, PKCI(3), PKCgamma, SCA14	protein kinase C gamma
5599	MAPK8	JNK, JNK-46, JNK1, JNK1A2, JNK21B1/2, PRKM8, SAPK1, SAPK1c	mitogen-activated protein kinase 8
5617	PRL	GHA1	prolactin
5625	PRODH	TP53I6, PRODH	proline dehydrogenase 1
5708	PSMD2	P97, RPN1, S2, TRAP2	proteasome 26S subunit ubiquitin receptor, non-ATPase 2
5724	PTAFR	PAFR	platelet activating factor receptor
5816	PVALB	D22S749	parvalbumin
5832	ALDH18A1	ADCL3, ARCL3A, GSAS, P5CS, PYCS, SPG9, SPG9A, SPG9B	aldehyde dehydrogenase 18 family member A1
5836	PYGL	GSD6	glycogen phosphorylase L
5859	QARS1	GLNRS, MSCCA, PRO2195, QARS	glutaminyl-tRNA synthetetase 1

GeneID	Symbol	Aliases	description
5864	RAB3A		RAB3A, member RAS oncogene family
5917	RARS1	ArgRS, DALRD1, HLD9, RARS	arginyl-tRNA synthetase 1
5923	RASGRF1	CDC25, CDC25L, GNRP, GRF1, GRF55, H-GRF55, PP13187, ras-GRF1	Ras protein specific guanine nucleotide releasing factor 1
5925	RB1	OSRC, PPP1R130, RB, p105-Rb, p110-RB1, pRb, pp110	RB transcriptional corepressor 1
5972	REN	ADTKD4, HNFJ2, RTD	renin
6132	RPL8	L8	ribosomal protein L8
6133	RPL9	L9, NPC-A-16	ribosomal protein L9
6135	RPL11	DBA7, GIG34, L11, uL5	ribosomal protein L11
6168	RPL37A	L37A	ribosomal protein L37a
6175	RPLP0	L10E, LP0, P0, PRLP0, RPP0	ribosomal protein lateral stalk subunit P0
6272	SORT1	Gp95, LDLCQ6, NT3, NTR3	sortilin 1
6285	S100B	NEF, S100, S100-B, S100beta	S100 calcium binding protein B
6311	ATXN2	ATX2, SCA2, TNRC13	ataxin 2
6347	CCL2	GDCF-2, HC11, HSMCR30, MCAF, MCP-1, MCP1, SCYA2, SMC-CF	C-C motif chemokine ligand 2
6390	SDHB	CWS2, IP, MC2DN4, PGL4, SDH, SDH1, SDH2, SDHIP	succinate dehydrogenase complex iron sulfur subunit B
6426	SRSF1	ASF, SF2, SF2p33, SFRS1, SRp30a	serine and arginine rich splicing factor 1
6443	SGCB	A3b, LGMD2E, LGMDR4, SGC	sarcoglycan beta
6506	SLC1A2	DEE41, EAAT2, EIEE41, GLT-1, HBGT	solute carrier family 1 member 2
6507	SLC1A3	EA6, EAAT1, GLAST, GLAST1	solute carrier family 1 member 3
6512	SLC1A7	AAAT, EAAT5	solute carrier family 1 member 7
6513	SLC2A1	GLUT, GLUT-1, GLUT1, GLUT1DS, HTLVR, PED, SDCHCN	solute carrier family 2 member 1
6517	SLC2A4	GLUT4	solute carrier family 2 member 4
6578	SLCO2A1	MATR1, OATP2A1, PGT, PHOAD, PHOAR2, SLC21A2	solute carrier organic anion transporter family member 2A1
6582	SLC22A2	oct-02	solute carrier family 22 member 2
6609	SMPD1	ASM, ASMASE, NPD	sphingomyelin phosphodiesterase 1
6610	SMPD2	ISC1, NSMASE, NSMASE1	sphingomyelin phosphodiesterase 2
6616	SNAP25	SNAP-25, SUP, bA416N4.2, dJ1068F16.2	synaptosome associated protein 25
6647	SOD1	ALS, ALS1, HEL-S-44, IPOA, SOD, STAHP, hSod1, homodimer	superoxide dismutase 1
6696	SPP1	BNSP, BSPI, ETA-1, OPN	secreted phosphoprotein 1
6712	SPTBN2	GTRAP41, SCA5, SCAR14	spectrin beta, non-erythrocytic 2
6750	SST	SMST1, SST	somatostatin
6853	SYN1	EPILX, MRX50a, SYN1b, SYNI, SYN1	synapsin I
6855	SYP	MRX96, MRXSYP, XLID96	synaptophysin

GeneID	Symbol	Aliases	description
6863	TAC1	Hs.2563, NK2, NKNA, NPK, TAC2	tachykinin precursor 1
6869	TACR1	NK1R, NKIR, SPR, TAC1R	tachykinin receptor 1
6874	TAF4	TAF2C1A, TAFII-130, TAFII-135, TAFII130, TAFII135, TAF4	TATA-box binding protein associated factor 4
7042	TGFB2	G-TSF, LDS4, TGF-beta2	transforming growth factor beta 2
7043	TGFB3	beta3	transforming growth factor beta 3
7047	TGM4	TGP, hTGP	transglutaminase 4
7051	TGM1	ARCI1, ICR2, KTG, LI, LI1, TGASE, TGK	transglutaminase 1
7052	TGM2	G(h), TG(C), TGC, hTG2, tTG	transglutaminase 2
7053	TGM3	TGE, UHS2	transglutaminase 3
7054	TH	DYT14, DYT5b, TYH	tyrosine hydroxylase
7056	THBD	AHUS6, BDCA-3, BDCA3, CD141, THPH12, THRM, TM	thrombomodulin
7086	TKT	HEL-S-48, HEL107, SDDHD, TK1, TKT	transketolase
7157	TP53	BCC7, BMFS5, LFS1, P53, TRP53	tumor protein p53
7276	TTR	ATTR, CTS, CTS1, HEL111, HsT2651, PALB, TBPA, TTN	transthyretin
7311	UBA52	CEP52, HUBCEP52, L40, RPL40	ubiquitin A-52 residue ribosomal protein fusion product 1
7341	SUMO1	DAP1, GMP1, OFC10, PIC1, SENP2, SMT3, SMT3C, SMT3H3, UBL1	small ubiquitin like modifier 1
7353	UFD1	UFD1L	ubiquitin recognition factor in ER associated degradation 1
7389	UROD	PCT, UPD	uroporphyrinogen decarboxylase
7415	VCP	CDC48, FTDALS6, TERA, p97	valosin containing protein
7422	VEGFA	MVCD1, VEGF, VPF	vascular endothelial growth factor A
7529	YWHAB	GW128, HEL-S-1, HS1, KCIP-1, YWHAA	tyrosine 3-monooxygenase/tryptophan 5-monooxygenase activation protein beta
7534	YWHAZ	14-3-3-zeta, HEL-S-3, HEL-S-93, HEL4, KCIP-1, POPCHAS, YWHAD	tyrosine 3-monooxygenase/tryptophan 5-monooxygenase activation protein zeta
7965	AIMP2	HLD17, JTV-1, JTV1, P38	aminoacyl tRNA synthetase complex interacting multifunctional protein 2
8106	PABPN1	OPMD, PAB2, PABII, PABP-2, PABP2	poly(A) binding protein nuclear 1
8140	SLC7A5	MPE16	solute carrier family 7 member 5
8202	NCOA3	ACTR, AIB-1, AIB1, CAGH16, CTG26, KAT13B, RAC3, SRC-3, SRC3, TNRC14, TNRC16, TRAM-1, bHLHe42, pCIP	nuclear receptor coactivator 3
8536	CAMK1	CAMKI	calcium/calmodulin dependent protein kinase I
8565	YARS1	YRS, YTS	tyrosyl-tRNA synthetase 1
8604	SLC25A12	AGC1, ARALAR, DEE39, EIEE39	solute carrier family 25 member 12
8704	B4GALT2	B4Gal-T2, B4Gal-T3, beta4Gal-T2	beta-1,4-galactosyltransferase 2

GeneID	Symbol	Aliases	description
8778	SIGLEC5	SIGLEC-5	sialic acid binding Ig like lectin 5
8942	KYNU	KYNUU, VCRL2	kynureninase
9001	HAP1	HAP2, HIP5, HLP, hHLP1	huntingtin associated protein 1
9020	MAP3K14	FTDCR1B, HS, HSNIK, NIK	mitogen-activated protein kinase kinase kinase 14
9120	SLC16A6	MCT6, MCT7	solute carrier family 16 member 6
9131	AIFM1	COXPD6, DFNX5, NADMR, NAMSD, PDCD8, SEMDHL	apoptosis inducing factor mitochondria associated 1
9159	PCSK7	LPC, PC7, PC8, SPC7	proprotein convertase subtilisin/kexin type 7
9255	AIMP1	EMAP2, EMAPII, HLD3, SCYE1, p43	aminoacyl tRNA synthetase complex interacting multifunctional protein 1
9333	TGM5	TGMX, TGX	transglutaminase 5
9365	KL	HFTC3A, KL	klotho
9410	SNRNP40	SPF38, WDR57	small nuclear ribonucleoprotein U5 subunit 40
9521	EEF1E1	AIMP3, P18	eukaryotic translation elongation factor 1 epsilon 1
9563	H6PD	CORTRD1, G6PDH, GDHH, H6PD	hexose-6-phosphate dehydrogenase/glucose 1-dehydrogenase
9759	HDAC4	AHO3, BDMR, HA6116, HD4, HDAC-4, HDAC-A, HDACA	histone deacetylase 4
10013	HDAC6	CPBHM, HD6, JM21, PPP1R90	histone deacetylase 6
10049	DNAJB6	LGMD1D, LGMD1E, LGMDD1, MRJ, MSJ-1	DnaJ heat shock protein family (Hsp40) member B6
10142	AKAP9	AKAP-9, AKAP350, AKAP450, CG-NAP, HYPERION, LQT11, MU-RMS-40.16A, PPP1R45, PRKA9, YOTIAO	A-kinase anchoring protein 9
10201	NME6	IPIA-ALPHA, NDK 6, NM23-H6	NME/NM23 nucleoside diphosphate kinase 6
10273	STUB1	CHIP, HSPABP2, NY-CO-7, SCA48, SCAR16, SDCCAG7, UBOX1	STIP1 homology and U-box containing protein 1
10280	SIGMAR1	BP, SR-BP1, SRBP, hSigmaR1, sigma1R	sigma non-opioid intracellular receptor 1
10458	BAIAP2	BAP2, FLAF3, IRSP53, WAML	BAR/IMD domain containing adaptor protein 2
10631	POSTN	OSF-2, OSF2, PDLPOSTN, PN	periostin
11113	CIT	CITK, CRIK, MCPH17, STK21	citron rho-interacting serine/threonine kinase
11318	GPR182	7TMR, ADMR, AM-R, AMR, G10D, L1-R, gamrh, hrhAMR	G protein-coupled receptor 182
22861	NLRP1	DEFCAP, DEFCAP-L/S, JRRP, MSPC, NAC, NALP1, PP1044, SLEV1, VAMAS1	NLR family pyrin domain containing 1
23007	PLCH1	PLC eta 1, PLC-L3, PLCL3	phospholipase C eta 1

GeneID	Symbol	Aliases	description
23236	PLCB1	DEE12, EIEE12, PI-PLC, PLC-154, PLC-I, PLC-beta-1, PLC154A, PLCB1B, PLCB1	phospholipase C beta 1
23435	TARDBP	ALS10, TDP-43	TAR DNA binding protein
23583	SMUG1	FDG, HMUDG, UNG3	single-strand-selective monofunctional uracil-DNA glycosylase 1
23657	SLC7A11	CCBR1, xCT	solute carrier family 7 member 11
26227	PHGDH	NLS1, PDG, PGAD, PGD, PGDHD, SERA, PHGDH	phosphoglycerate dehydrogenase
26762	HAVCR1	KIM1, TIM, TIM-1, TIM1, TIMD-1, TIMD1	hepatitis A virus cellular receptor 1
27036	SIGLEC7	siglec, QA79, SIGLEC-7, SIGLEC19P, SIGLECP2, p75, p75/AIRM1	sialic acid binding Ig like lectin 7
29803	REPIN1	AP4, RIP60, ZNF464, Zfp464	replication initiator 1
29968	PSAT1	EPIP, NLS2, PSA, PSAT, PSATD	phosphoserine aminotransferase 1
29978	UBQLN2	N4BP4, PLIC2	ubiquilin 2
50489	CD207	CLEC4K	CD207 molecule
51143	DYNC1LI1	DLC-A, DNCLI1, LIC1	dynein cytoplasmic 1 light intermediate chain 1
51144	HSD17B12	KAR, SDR12C1	hydroxysteroid 17-beta dehydrogenase 12
51340	CRNKL1	CLF, CRN, Clf1, HCRN, MSTP021, SYF3	crooked neck pre-mRNA splicing factor 1
51520	LARS1	HSPC192, ILFS1, LARS, LEURS, LEUS, LFIS, LRS, PIG44, RNTLS, hr025Cl	leucyl-tRNA synthetase 1
54205	CYCS	CYC, HCS, THC4	cytochrome c, somatic
54407	SLC38A2	ATA2, PRO1068, SAT2, SNAT2	solute carrier family 38 member 2
55512	SMPD3	NSMASE2	sphingomyelin phosphodiesterase 3
55922	NKRF	ITBA4, NRF	NFKB repressing factor
56985	ADPRM	C17orf48, MDS006, NBLA03831	ADP-ribose/CDP-alcohol diphosphatase, manganese dependent
57026	PDXP	CIN, PLP, dJ37E16.5	pyridoxal phosphatase
57380	MRS2	HPTL, MRS2	magnesium transporter MRS2
57674	RNF213	ALO17, C17orf27, KIAA1618, MYMY2, MYSTR, NET57	ring finger protein 213
57704	GBA2	AD035, NLGase, SPG46	glucosylceramidase beta 2
64802	NMNAT1	LCA9, NMNAT, PNAT1, SHILCA	nicotinamide nucleotide adenylyltransferase 1
80150	ASRGL1	ALP, ALP1, CRASH	asparaginase and isoaspartyl peptidase 1
81539	SLC38A1	ATA1, NAT2, SAT1, SNAT1	solute carrier family 38 member 1
84148	KAT8	hMOF	lysine acetyltransferase 8
84706	GPT2	ALT2, GPT 2, MRT49, NEDSPM	glutamic--pyruvic transaminase 2
84991	RBM17	SPF45	RNA binding motif protein 17
90678	LRSAM1	CMT2P, RIFLE, TAL	leucine rich repeat and sterile alpha motif containing 1
93986	FOXP2	CAGH44, SPCH1, TNRC10	forkhead box P2

GeneID	Symbol	Aliases	description
114757	CYGB	HGB, STAP	cytoglobin
116179	TGM7	TGMZ	transglutaminase 7
116443	GRIN3A	GluN3A, NMDAR-L, NMDAR3A, NR3A	glutamate ionotropic receptor NMDA type subunit 3A
116444	GRIN3B	GluN3B, NR3B	glutamate ionotropic receptor NMDA type subunit 3B
117247	SLC16A10	MCT10, PRO0813, TAT1	solute carrier family 16 member 10
140738	TMEM37	PR, PR1	transmembrane protein 37
146713	RBFOX3	FOX-3, FOX3, HRNBP3, NEUN	RNA binding fox-1 homolog 3
166929	SGMS2	CDL, SMS2	sphingomyelin synthase 2
197257	LDHD	DLACD, DLD	lactate dehydrogenase D
283464	GXYLT1	GLT8D3	glucoside xylosyltransferase 1
343641	TGM6	SCA35, TG6, TGM3L, TGY, dJ734P14.3	transglutaminase 6
